# Sleep deprivation increases formation of false memory

**DOI:** 10.1111/jsr.12436

**Published:** 2016-07-05

**Authors:** June C. Lo, Pearlynne L. H. Chong, Shankari Ganesan, Ruth L. F. Leong, Michael W. L. Chee

**Affiliations:** ^1^ Centre for Cognitive NeuroscienceNeuroscience and Behavioral Disorders ProgramDuke‐NUS Medical SchoolSingapore

**Keywords:** adolescents, adults, cognitive function, false memory, memory formation, sleep deprivation

## Abstract

Retrieving false information can have serious consequences. Sleep is important for memory, but voluntary sleep curtailment is becoming more rampant. Here, the misinformation paradigm was used to investigate false memory formation after 1 night of total sleep deprivation in healthy young adults (*N* = 58, mean age ± SD = 22.10 ± 1.60 years; 29 males), and 7 nights of partial sleep deprivation (5 h sleep opportunity) in these young adults and healthy adolescents (*N* = 54, mean age ± SD = 16.67 ± 1.03 years; 25 males). In both age groups, sleep‐deprived individuals were more likely than well‐rested persons to incorporate misleading post‐event information into their responses during memory retrieval (*P *< 0.050). These findings reiterate the importance of adequate sleep in optimal cognitive functioning, reveal the vulnerability of adolescents' memory during sleep curtailment, and suggest the need to assess eyewitnesses' sleep history after encountering misleading information.

## Introduction

Memories of an event rarely provide a literal record of that experience. Instead, they involve the integration of elements of that episode with prior experience or knowledge. A highly novel or distinct experience, for example a first publication in a high‐impact journal, is rarely mis‐remembered. However, when the memory of a specific episode is confused with prior similar experiences, and/or fails to be distinctly encoded, errors in subsequent memory retrieval can occur. The emergence of such false memories often reminds us of human fallibility, as highlighted by the inconsistencies in recollection of personal events surrounding the Challenger disaster (Neisser and Harsch, 1992). However, they can also have more serious consequences such as wrongful conviction due to inaccurate eyewitness testimony.

Adequate sleep is essential to optimize memory processes (Diekelmann and Born, 2010; Stickgold and Walker, 2013). This is consistent across a range of tests evaluating veridical memory (Lo *et al*., 2014a; Payne *et al*., 2012; Rasch *et al*., 2007; Tamminen *et al*., 2010). However, relatively little attention has been paid to the possible effects of inadequate sleep on the formation of false memory. This is increasingly relevant because voluntary sleep curtailment, in young persons, has become widespread in developed societies (Steptoe *et al*., 2006).

Two paradigms are widely used in the laboratory to induce false memory. In the Deese–Roediger–McDermott (Roediger and McDermott, 1995) paradigm, participants learn lists of semantically related words, with words in each list sharing a common theme word that is never presented. Retrieval of these theme words (a measure of false memory) is affected by sleep, although the specific mechanism appears to vary with the retrieval strategy used (Diekelmann *et al*., 2008, 2010; Fenn *et al*., 2009; Lo *et al*., 2014b; McKeon *et al*., 2012; Payne *et al*., 2009).

The misinformation paradigm (Loftus *et al*., 1978; Okado and Stark, 2005) is another tool used to assess false memory. It involves inducing retroactive interference through the introduction of misleading information related to previously witnessed events. Misleading a person using this technique is ecologically more relevant to forensic and medical situations where the retrieval of critical details of an event can be disturbed by posing leading questions (Frenda *et al*., 2011). Using this paradigm, Frenda *et al*. (2014) found that 1 night of total sleep deprivation (TSD) elevated false memory formation in young adults. False memory formation also tended to be more prevalent in individuals reporting short versus long sleep duration on the night prior to the experiment, suggesting a possible effect of partial sleep deprivation (PSD). The latter point was clarified in Experiment 1 by investigating the effect of PSD on false memory formation in young adults. Effect size was also compared with that of TSD.

In some countries, >90% of adolescents sleep less than the recommended 8–10 h (Do *et al*., 2013; Ohida *et al*., 2004). Despite the well‐documented degradation of sustained attention, working memory and executive functions in young adults undergoing PSD (Lo *et al*., 2012; Van Dongen *et al*., 2003), experimental studies in adolescents suggest that the negative cognitive consequences of PSD are milder in this age group (Carskadon *et al*., 1981; Fallone *et al*., 2001; Kopasz *et al*., 2010). For example, even when adolescents were restricted to 5 h sleep opportunity for 4 nights, several cognitive functions, including veridical recall of declarative memory, were spared, leading some to suggest that adolescents may be resilient to sleep restriction (Voderholzer *et al*., 2011). To test this proposal, in Experiment 2, the effect of PSD on the formation of veridical as well as false memory formation in adolescents was evaluated.

## Materials and methods

### Experiment 1

#### Participants

Sixty healthy undergraduate students, who did not report any symptoms of sleep apnea (the Berlin Questionnaire; Netzer *et al*., 1999), exhibit extreme morningness–eveningness preference [the Morningness–Eveningness Questionnaire (MEQ); Horne and Ostberg, 1976], or consume >5 cups of caffeinated beverages each day, were randomized into three groups: control group; PSD group; and TSD group. Two TSD participants failed to adhere to the assigned sleep schedule and were excluded from all analyses. Thus, the final sample included 58 participants (mean age ± SD = 22.10 ± 1.60 years; 29 males). The three groups were comparable in age, gender distribution, MEQ score and self‐reported habitual sleep duration (*P *> 0.344; Table 1).

**Table 1:** Sample characteristics of Experiment 1

	Control group		PSD group		TSD group		*F*/χ^2^	*P*
	Mean	SEM	Mean	SEM	Mean	SEM		
*n*	20	–	20	–	18	–	–	–
Age (years)	22.50	0.32	21.90	0.37	21.89	0.41	0.93	0.402
Gender (% males)	50.00	–	50.00	–	50.00	–	0.00	0.999
Morningness–Eveningness Questionnaire score	46.65	1.89	50.11	1.87	47.72	1.83	0.91	0.407
Self‐reported habitual sleep duration (h)	7.03	0.22	6.65	0.28	6.50	0.28	1.09	0.344

PSD, partial sleep deprivation; SEM, standard error of the mean; TSD, total sleep deprivation.

#### Design and procedure

**Figure 1:**
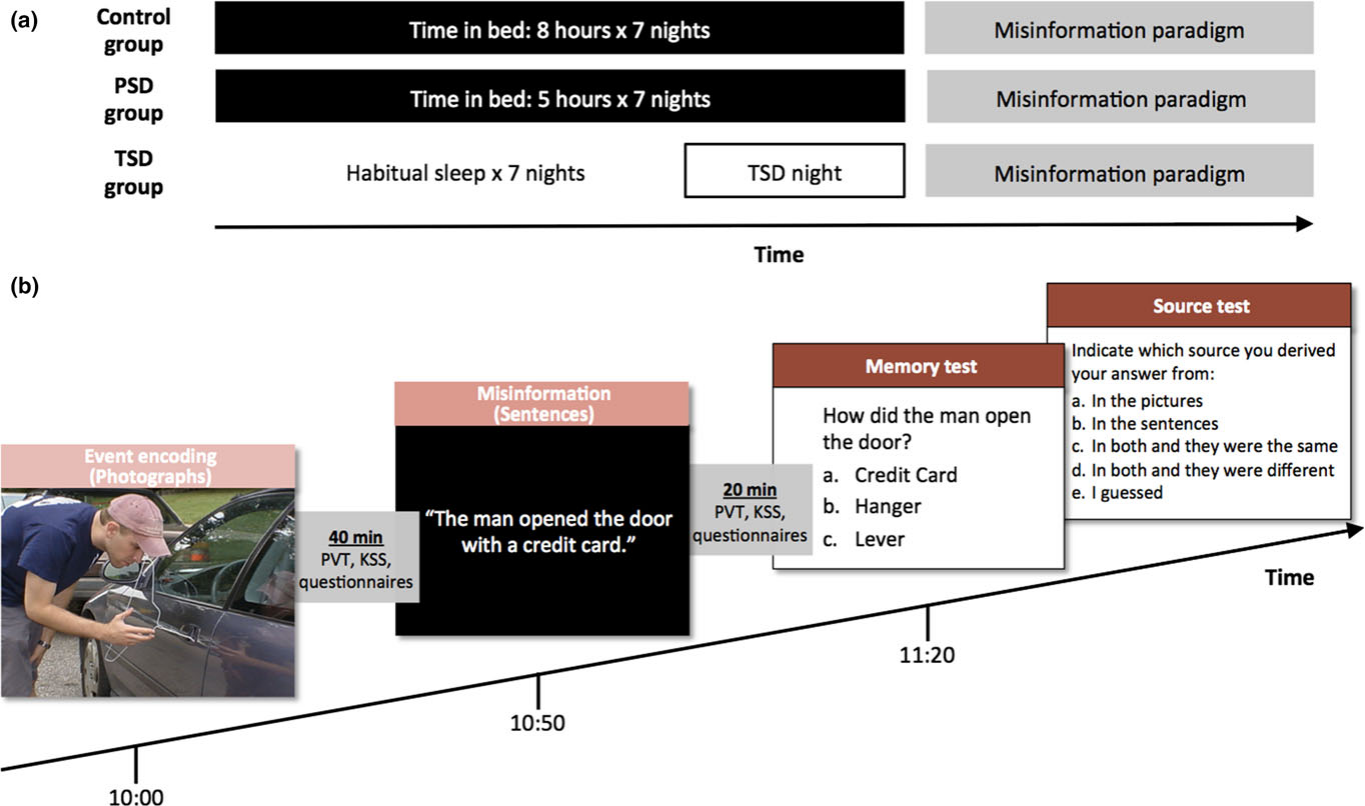
Protocol of Experiment 1. (a) The three groups of participants differed in their sleep history prior to performing the misinformation paradigm. While time in bed (TIB) for the control and the partial sleep deprivation (PSD) groups were 8 h and 5 h, respectively, for 7 nights, the total sleep deprivation (TSD) group followed their habitual sleep schedule for 7 nights before spending an entire night awake at the laboratory. (b) The misinformation paradigm was administered at 10:00 hours after the sleep history manipulation period. Participants were shown two crimes in the forms of photographs (event‐encoding phase) and narratives that might not be consistent with the photographs (misinformation phase). Memory of the crimes was tested in the third phase (memory and source tests). Successive phases of the misinformation paradigm were, respectively, separated by a 40‐min and a 20‐min period during which participants completed the Psychomotor Vigilance Task (PVT), the Karolinska Sleepiness Scale (KSS), and some questionnaires.

This study employed a between‐subject design (Fig. 1a). The control and PSD groups were required to stay in bed for 8 h and 5 h each night, respectively, for 7 nights prior to the experiment session. On the day of the experiment, these participants were instructed to wake up by 09:00 hours. The TSD group followed their habitual sleep schedule for 7 nights, and in the morning prior to the TSD night woke up by 09:00 hours and stayed awake until 10:00 hours the following day. From 20:00 hours to 10:00 hours, they were under constant supervision by research staff in the laboratory, which had natural and artificial lighting, and caffeine‐free snacks were provided if necessary. Daytime naps were not permitted during the 1‐week period of sleep manipulation. Compliance with sleep schedules was verified with actigraphy (Actiwatch 2, Respironics). Consumption of caffeinated beverages was prohibited 24 h prior to the experiment session. The misinformation experiment commenced at 10:00 hours after the last manipulation night.

This study was approved by the Institutional Review Board of the National University of Singapore, and conducted in accordance with the provisions of the Declaration of Helsinki. All the participants provided written informed consent.

#### Materials

##### Misinformation paradigm

The misinformation paradigm (Okado and Stark, 2005) consisted of three phases: event‐encoding; misinformation; and memory and source tests (Fig. 1b). In the event‐encoding phase, two sets of 50 photographs depicting two crimes (a car break‐in and a robbery) were presented. Each photograph was shown for 3500 ms with an inter‐stimulus interval of 500 ms. Participants were instructed to pay close attention to the photographs as they might be questioned on them.

Forty minutes after the event‐encoding phase, participants entered the misinformation phase. Two sets of 50 narratives were presented, with each corresponding to a previously shown photograph. Each narrative sentence was presented for 5500 ms with an inter‐stimulus interval of 500 ms. For each crime, 12 of the narratives contradicted the content of the photograph. Participants were therefore exposed to 24 pieces of misinformation regarding central details of the event, interleaved with sentences containing information that was consistent with the photographs. Participants were asked to pay close attention to the narratives and told that they might be questioned on them later, but were not informed of the discrepancies between the photographs and the narratives.

Memory of the two crimes was assessed 20 min after the misinformation phase. The memory test consisted of 36 three‐alternative forced‐choice questions, and participants were instructed to answer based on their knowledge of ‘the photographs alone’. For each crime, 12 of the 18 questions were critical questions and probed memory of items with discrepancies between the photographs and narratives. The other six questions were non‐critical questions involving memoranda that were consistent across photographs and narratives. Among the three alternatives for the critical questions, one was a novel foil, one was the correct answer (consistent with the photograph), and one was consistent with the misinformation presented in the narrative. For the non‐critical questions, an additional novel foil was present instead of any misinformation. In the source memory test, participants were shown previously answered questions together with their answers, and had to indicate if their answer was based on: ‘in the pictures only’, ‘in the narratives only’, ‘in both and they were the same’, ‘in both and they were different’, or ‘I guessed’.

Three measures of performance were derived. The correct memory rate, a measure of veridical memory, was defined as the percentage of non‐critical questions to which participants provided the correct answer. There were two measures of false memory. The misinformation consistent response rate was the percentage of critical questions for which participants incorporated misinformation from the narratives into their responses in the memory test. The false memory rate was the percentage of critical questions for which participants both incorporated misinformation from the narratives into their responses, and misattributed the source of information as a photograph.

##### Psychomotor Vigilance Task (PVT)

A 10‐min PVT (Dinges and Powell, 1985) was administered to measure sustained attention between successive phases of the misinformation paradigm. A counter on the computer screen started counting at random intervals between 2 and 10 s. Participants were required to respond as quickly as possible. Performance was indicated by the number of lapses (responses exceeding 500 ms).

##### Karolinska Sleepiness Scale (KSS)

The KSS (Akerstedt and Gillberg, 1990) was used to measure levels of subjective sleepiness after the event‐encoding phase and the misinformation phase. Participants chose one of nine levels of sleepiness (1 = ‘extremely alert’; 3 = ‘alert’; 5 = ‘neither sleepy nor alert’; 7 = ‘sleepy but not fighting sleep’; 9 = ‘extremely sleepy, it is an effort to stay awake’) that best described their current level of sleepiness. Participants also completed other questionnaires not pertinent to the present report.

#### Statistical analyses

Statistical analyses were conducted using SPSS Version 21 (IBM, Chicago, IL, USA). One‐way anovas and independent‐samples *t*‐tests were used to determine group differences in cognitive performance and the KSS scores. The effect sizes of PSD and TSD (relative to the control group) on performance in the misinformation paradigm were quantified using Cohen's *d*. The conventional cut‐offs for small, medium and large effect sizes are 0.20, 0.50 and 0.80, respectively (Cohen, 1988).

### Experiment 2

#### Participants

Sixty secondary school students participated in the Need for Sleep Study – a 2‐week protocol that examined changes in cognitive performance, subjective sleepiness and mood during PSD. Subjects were 15–19 years old and healthy; they were not habitual short sleepers and did not consume >5 cups of caffeinated beverages each day (for more details of study implementation and selection criteria, see Lo *et al*., 2016). Participants were randomized into control and PSD groups. Because of withdrawals (*n* = 3), non‐compliance with the experimental procedures (*n* = 1) and technical errors (*n* = 2), the final sample included 54 participants (mean age ± SD = 16.67 ± 1.03 years; 25 males). The two groups did not differ in age, gender distribution, body mass index, MEQ score or self‐reported habitual sleep duration (*P *> 0.166; Table 2).

**Table 2:** Sample characteristics of Experiment 2

	Control group		PSD group		*t*/χ^2^	*P*
	Mean	SEM	Mean	SEM		
*n*	25	–	29	–		
Age (years)	16.88	0.23	16.48	0.17	1.43	0.166
Gender (% males)	44.00	–	48.28	–	0.10	0.790
Body mass index	20.52	0.50	20.40	0.54	0.16	0.871
Morningness–Eveningness Questionnaire score	49.84	1.45	47.93	1.40	0.94	0.351
Self‐reported habitual sleep duration (h)	6.86	0.15	6.96	0.13	0.49	0.627

PSD, partial sleep deprivation; SEM, standard error of the mean.

#### Design and procedure

**Figure 2:**
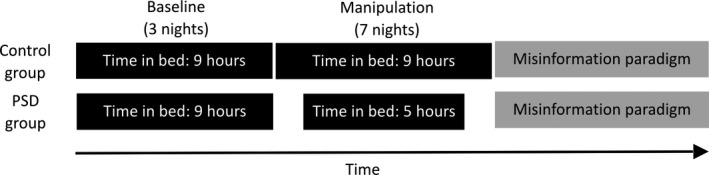
Protocol of Experiment 2. Both the control and the partial sleep deprivation (PSD) groups had three baseline nights of 9 h time in bed (TIB), followed by a manipulation period of 7 nights, when TIB was reduced to 5 h for the PSD group but remained at 9 h for the control group. After the manipulation period, the misinformation paradigm was administered.

This study employed a between‐subject design (Fig. 2). One week prior to this 2‐week protocol, participants followed a 9‐h sleep schedule at home (23:00–08:00 hours), which was verified with actigraphy (Actiwatch 2, Respironics). The protocol began with three baseline nights of 9 h time in bed (TIB; 23:00–08:00 hours), followed by 7 nights of sleep opportunity manipulation [5 h TIB (01:00–06:00 hours) and 9 h TIB (23:00–08:00 hours) for the PSD and control groups, respectively], and ended with three recovery nights of 9 h TIB (23:00–08:00 hours). Sleep duration and macrostructure were measured with polysomnography (PSG) on the first, fourth and seventh manipulation night (M1, M4 and M7).

The misinformation paradigm, identical to that used in Experiment 1, was administered at 14:00 hours after the last night of sleep opportunity manipulation. The event‐encoding phase was followed by a 40‐min period when participants completed a cognitive test battery, inclusive of the PVT and the KSS. The misinformation phase was then administered, followed by a 20‐min period when participants completed the KSS and some questionnaires not pertinent to the current report. Afterwards, participants completed a memory test and a source test.

This study was approved by the Institutional Review Board of the National University of Singapore, and conducted in accordance with the provisions of the Declaration of Helsinki. All participants and their parent or guardian provided written informed consent.

#### PSG

Electroencephalogram (EEG) was recorded using a SOMNOtouch recorder (SOMNOmedics GmbH, Randersacker, Germany) from two channels (C3 and C4 in the international 10–20 system) referenced to the contralateral mastoids. The common ground and reference electrodes were placed at Cz and FPz respectively. Electrooculography (EOG) and submental electromyography (EMG) were also used. Impedance was kept below 5 kΩ for EEG electrodes, and below 10 kΩ for EOG and EMG electrodes. Signals were sampled at 256 Hz, and filtered between 0.2 and 35 Hz for EEG and between 0.2 and 10 Hz for EOG.

Sleep scoring analyses were performed using the FASST toolbox (http://www.montefiore.ulg.ac.be/~phillips/FASST.html). EEG signals were band‐pass filtered between 0.2 and 25 Hz. Scoring was performed visually by trained technicians following the criteria set by the AASM Manual for the Scoring of Sleep and Associated Events (Iber *et al*., 2007). Total Sleep Time (TST) and sleep macrostructure, i.e. time spent in different sleep stages, were determined for the beginning, middle and end of the sleep opportunity manipulation period (nights M1, M4 and M7).

#### Statistical analyses

Independent‐samples *t*‐tests were used to determine group differences in performance, KSS score and sleep. To determine the contribution of sleep in the night immediately before the misinformation paradigm, and the cumulative effects of sleep loss during the manipulation period to false memory formation, PSG analyses focused on sleep parameters for the last manipulation night and the average across the three PSG‐recorded manipulation nights. Pearson correlations were used to examine the associations between sleep and false memory formation.

## Results

### Experiment 1

#### Actigraphically assessed sleep during the manipulation period

The average TIB for the PSD group was 5.08 h (SEM = 0.08 h), which was significantly shorter than the 7.89 h TIB (SEM = 0.04 h) of the control group (*t*_38_ = 32.57, *P *< 0.001). Similarly, TST was also shorter for the PSD than the control groups (4.67 ± 0.11 h versus 6.92 ± 0.14 h, *t*_38_ = 12.63, *P *< 0.001). The TIB and TST of the TSD group were 6.93 h (SEM = 0.22 h) and 6.17 h (SEM = 0.23 h), respectively, indicating that these participants did not lengthen their sleep prior to the night of TSD.

#### PVT and KSS

The number of lapses did not significantly differ across the three groups in the PVT administered after the event‐encoding phase (*F*_2,55_ = 1.23, *P* = 0.300; Table 3), but a significant group difference was found after the misinformation phase (*F*_2,55_ = 4.01, *P* = 0.024). *Post hoc* independent‐samples *t*‐tests revealed that while the PSD and TSD groups did not differ in the number of lapses (*t*_36_ = 0.59, *P* = 0.560), these two groups had more lapses than the control group (PSD versus control: *t*_38_ = 2.49, *P* = 0.022; TSD versus control: *t*_36_ = 3.49, *P* = 0.002).

**Table 3:** Performance in the PVT and the KSS score in Experiment 1

	Control group		PSD group		TSD group		*F*	*P*
	Mean	SEM	Mean	SEM	Mean	SEM		
PVT number of lapses								
After event‐encoding	1.65	0.59	6.20	2.99	3.94	1.29	1.23	0.300
After misinformation	1.90* ^,^ †	0.63	10.85*	3.54	8.44†	1.77	4.01	0.024
KSS score								
After event‐encoding	4.45* ^,^ †	0.43	6.30*	0.39	6.67†	0.44	8.16	<0.001
After misinformation	5.00* ^,^ †	0.45	7.10*	0.36	7.17†	0.46	8.61	<0.001

*^,†^Indicate significant contrasts of the PSD and TSD groups relative to the control group, respectively.

KSS, Karolinska Sleepiness Scale; PSD, partial sleep deprivation; PVT, Psychomotor Vigilance Task; SEM, standard error of the mean; TSD, total sleep deprivation.

Partial sleep deprivation and TSD increased levels of subjective sleepiness. A significant group difference in KSS scores was found both after the event‐encoding phase and the misinformation phase (*F*_2,55_ = 8.16 and 8.61, *P *< 0.001), with the PSD group (*t*_38_ = 3.22, *P* = 0.003; *t*_38_ = 3.65, *P *< 0.001) and the TSD group (*t*_36_ = 3.61, *P* = 0.001; *t*_36_ = 3.35, *P* = 0.002; Table 3) reporting being sleepier than the control group. KSS scores were similar between the TSD and PSD groups (*t*_36_ = 0.63, *P* = 0.532; *t*_36_ = 0.12, *P* = 0.904; Table 3).

#### Misinformation paradigm

**Figure 3:**
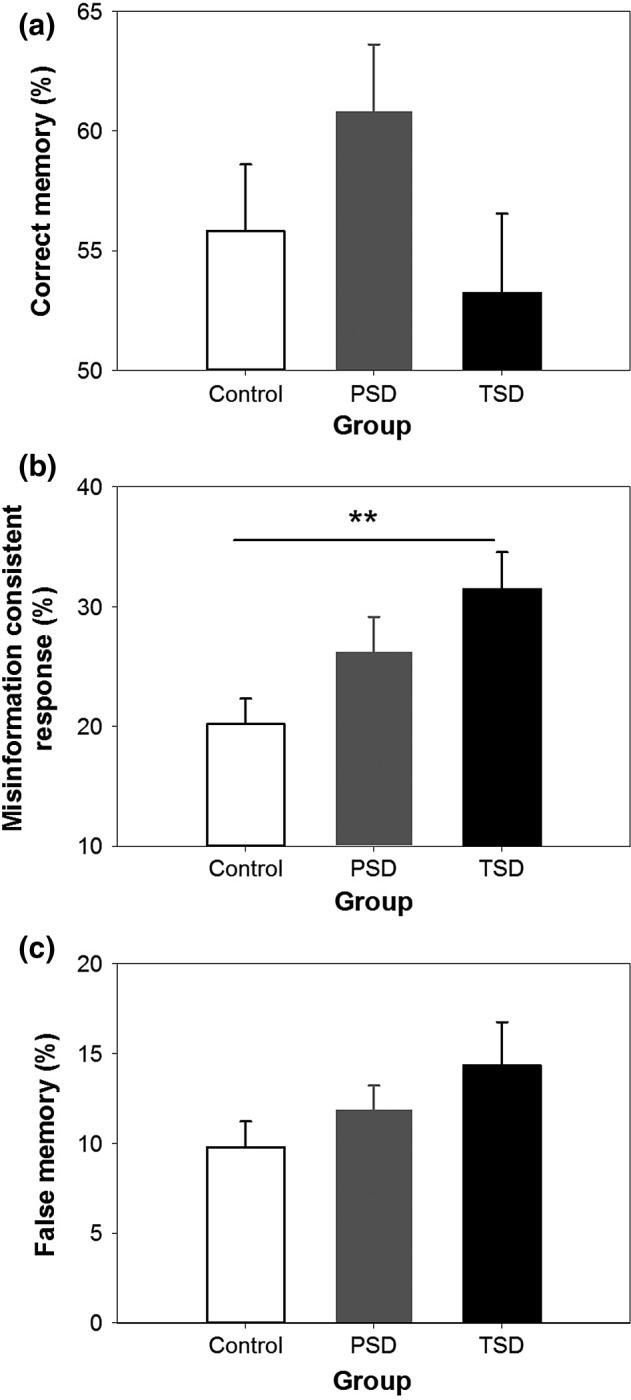
Performance in the misinformation paradigm in Experiment 1. Mean ± SEM of (a) correct memory rate, (b) misinformation consistent response rate, and (c) false memory rate of the control group (white bar), the partial sleep deprivation (PSD) group (grey bar), and the total sleep deprivation (TSD) group (black bar).

The three groups did not differ significantly in the correct memory rate (*F*_2,55_ = 1.71, *P* = 0.191; Fig. 3a). Hence, neither PSD nor TSD affected the formation of veridical memory.

The misinformation consistent response rate increased from the control group to the PSD group and the TSD group (*F*_2,55_ = 4.27, *P* = 0.019; Fig. 3b). *Post hoc* independent‐samples *t‐*tests revealed that the misinformation consistent response rate was significantly higher in the TSD group than in the control group (*t*_36_ = 3.07, *P* = 0.004). TSD had a large effect on this false memory measure (Cohen's *d* = 0.99). In contrast, the difference between the PSD and the control groups did not reach statistical significance (*t*_38_ = 1.68, *P* = 0.101), although the size of this PSD effect was in the medium range (*d* = 0.53).

To determine whether lower levels of vigilance and higher levels of subjective sleepiness in the TSD group relative to the control group contributed to the group difference in the misinformation consistent response rate, three ancova analyses were performed. Upon partialling out the group difference in sustained attention after the misinformation phase, and subjective sleepiness after the event‐encoding phase and the misinformation phase separately, the misinformation consistent response rate remained significantly higher in the TSD group (*F*_1,35_ = 6.38, *P* = 0.016; *F*_1,35_ = 11.88, *P* = 0.001; *F*_1,35_ = 9.20, *P* = 0.005). This suggests that the higher misinformation consistent response rate after a night of TSD could not be attributed to impaired sustained attention or subjective alertness.

In 10–15% of the critical questions, participants incorporated misinformation from the narratives into their responses, and misattributed the source of information as a photograph. Although there was a trend in the expected direction, the higher false memory rate in the PSD and TSD groups did not reach significance when compared with the control group (*F*_2,55_ = 1.70, *P* = 0.192; Fig. 3c).

### Experiment 2

#### PSG‐assessed sleep during the manipulation period

The PSD group had significantly shorter TST than the control group on M7 and throughout the sleep opportunity manipulation period (*t*_52_ = 50.72 and 61.70, *P *< 0.001; Table 4). Stage N3 duration was similar between the two groups (*P* = 0.095 and *P* = 0.572), but the duration of stage N1, N2 and rapid eye movement sleep was reduced in the PSD group (*P *< 0.001; Table 4).

**Table 4:** TST and sleep macrostructure in Experiment 2

	Control group		PSD group		*t*_52_	*P*
	Mean	SEM	Mean	SEM		
TST (min)						
M7	490.00	3.85	288.22	5.27	50.72	<0.001
M1, M4, M7 average	485.92	3.10	284.25	1.05	61.70	<0.001
N1 sleep (min)						
M7	16.10	1.23	3.46	0.43	9.69	<0.001
M1, M4, M7 average	16.20	1.29	4.71	0.48	8.32	<0.001
N2 sleep (min)						
M7	260.06	6.33	128.02	3.25	18.55	<0.001
M1, M4, M7 average	256.99	4.84	129.43	2.97	22.47	<0.001
N3 sleep (min)						
M7	98.19	5.54	109.26	17.29	1.71	0.095
M1, M4, M7 average	98.85	4.97	102.16	3.03	0.57	0.572
Non‐rapid eye movement sleep (min)						
M7	374.35	5.17	240.74	3.02	22.31	<0.001
M1, M4, M7 average	372.04	4.15	236.30	2.66	28.31	<0.001
Rapid eye movement sleep (min)						
M7	115.65	4.84	47.48	3.09	12.14	<0.001
M1, M4, M7 average	113.88	3.73	47.95	2.73	14.26	<0.001

M1, M4 and M7 represent the first, fourth and seventh nights during the sleep opportunity manipulation period, respectively.

PSD, partial sleep deprivation; SEM; standard error of the mean; TST, total sleep time.

#### PVT and KSS

The PSD group showed more PVT lapses than the control group (*t*_52_ = 7.00, *P *< 0.001; Table 5), revealing the impairing effect of PSD on sustained attention. The PSD group reported being sleepier than the control group, as evidenced by a higher KSS score both after the event‐encoding phase and the misinformation phase (*t*_50_ = 5.01 and 4.68, *P *< 0.001; Table 5).

**Table 5:** Performance in the PVT and the KSS score in Experiment 2

	Control group		PSD group		*t*	*P*
	Mean	SEM	Mean	SEM		
PVT number of lapses						
After event‐encoding	2.60	0.73	20.48	2.45	7.00	<0.001
KSS score						
After event‐encoding	5.75	0.34	7.82	0.25	5.01	<0.001
After misinformation	5.80	0.32	7.82	0.30	4.68	<0.001

KSS, Karolinska Sleepiness Scale; PSD, partial sleep deprivation; PVT, Psychomotor Vigilance Task; SEM, standard error of the mean.

#### Misinformation paradigm

**Figure 4:**
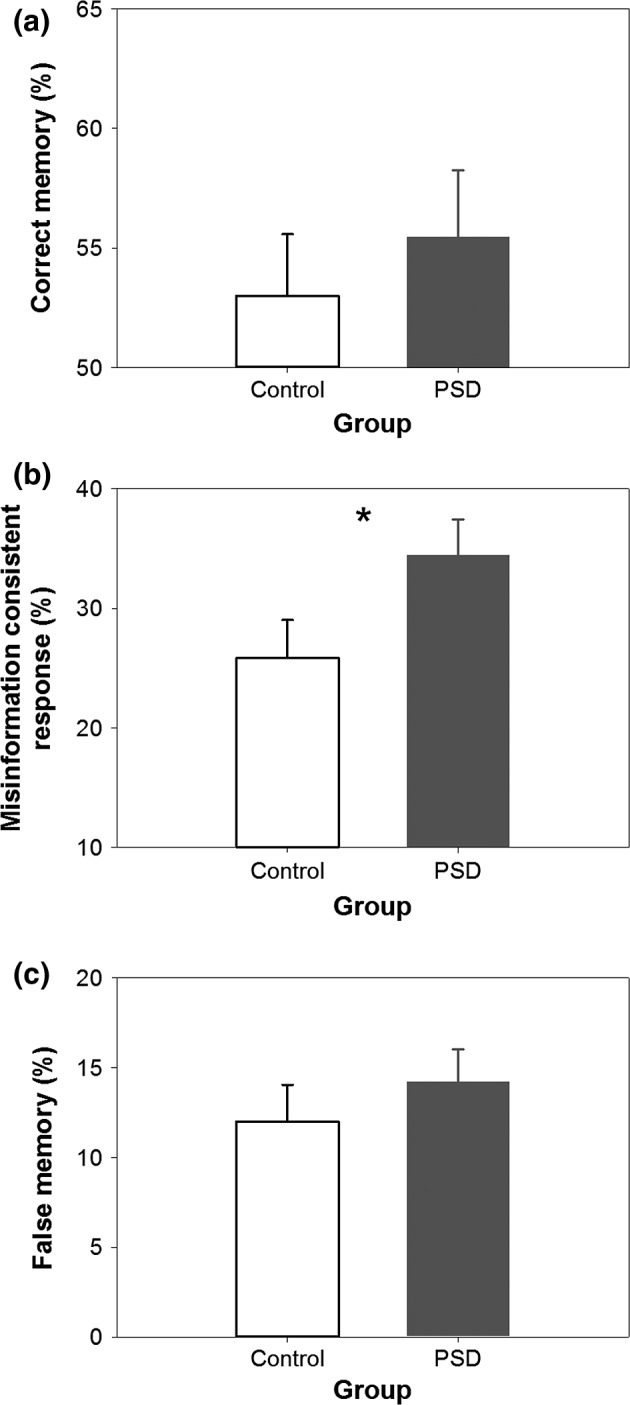
Performance in the misinformation paradigm in Experiment 2. Mean ± SEM of (a) correct memory rate, (b) misinformation consistent response rate, and (c) false memory rate of the control group (white bar) and the partial sleep deprivation (PSD) group (grey bar).

The control and PSD groups did not differ significantly in the correct memory rate (*t*_52_ = 0.64, *P* = 0.525; Fig. 4a). Hence, PSD did not affect the formation of veridical memory.

The misinformation consistent response rate was significantly higher in the PSD than in the control group (*t*_52_ = 2.01, *P *< 0.050; Fig. 4b). This PSD effect was in the medium range (*d* = 0.58). To determine whether this group difference was associated with the detrimental effects of PSD on sustained attention and subjective alertness, three ancova analyses were performed. After partialling out the effect of PSD on the number of PVT lapses, this false memory measure was no longer elevated in the PSD group (*F*_1,51_ = 2.38, *P* = 0.129), suggesting that poorer sustained attention induced by PSD might contribute to the higher misinformation consistent response rate. Upon partialling out the effect of PSD on KSS score after the event‐encoding phase and the misinformation phase, respectively, the misinformation consistent response rate remained significantly higher in the PSD group (*F*_1,49_ = 5.03, *P* = 0.029; *F*_1,51_ = 7.87, *P* = 0.007). Hence, higher levels of subjective sleepiness in a sleep‐deprived state could not account for the elevation in false memory formation.

The two groups did not significantly differ in their false memory rate (*t*_52_ = 0.82, *P* = 0.416; Fig. 4c), although as in the young adults, this rate was in the anticipated direction.

#### Correlation with sleep macrostructure

Partial sleep deprivation shortened TST and altered sleep macrostructure (Table 4), which might account for the increased misinformation consistent response rate in the PSD group. Pearson correlations were used to examine the linear associations between sleep and this false memory measure. However, because of the discontinuous nature of these sleep variables (Fig. S1), correlational analyses could not be conducted with the PSD and the control groups combined.

No significant association was found between misinformation consistent response rate and sleep parameters in the last manipulation night for either the control or the PSD group (*P *> 0.060; Table 6). Similarly, no significant relationships were found between these measures across the entire sleep opportunity manipulation period (*P *> 0.142; Table 6). These null findings could be due to the limitation of range restriction and the smaller sample sizes when analyses were performed separately for each group.

**Table 6:** Correlations between misinformation consistent response rate and sleep

	Control group		PSD group	
	*r*	*P*	*r*	*P*
TST				
M7	−0.20	0.337	0.37	0.060
M1, M4, M7 average	−0.18	0.396	0.17	0.369
Stage N1				
M7	−0.04	0.855	0.18	0.379
M1, M4, M7 average	0.02	0.932	−0.04	0.829
Stage N2				
M7	0.01	0.953	−0.32	0.099
M1, M4, M7 average	−0.17	0.414	−0.28	0.142
Stage N3				
M7	−0.06	0.770	0.25	0.218
M1, M4, M7 average	0.09	0.681	0.27	0.158
Stage non‐rapid eye movement				
M7	−0.06	0.775	−0.05	0.790
M1, M4, M7 average	−0.09	0.668	−0.01	0.948
Stage rapid eye movement				
M7	−0.10	0.651	0.17	0.388
M1, M4, M7 average	−0.05	0.823	0.08	0.683

M1, M4 and M7 represent the first, fourth and seventh sleep opportunity manipulation nights, respectively.

PSD, partial sleep deprivation; TST, total sleep time.

## Discussion

Using the misinformation paradigm, it was found that partially sleep‐deprived adolescents were more likely to incorporate misleading post‐event information into their responses while retrieving memories of the original event. This PSD effect in adolescents was of comparable magnitude to that observed in young adults. In young adults, the TSD effect was more prominent than the effect of PSD. In contrast to these robust effects on false memory, PSD and TSD did not appear to affect veridical memory.

The TSD effect in young adults parallels the finding of elevated false memory formation after a night of total sleep loss (Frenda *et al*., 2014). Additionally, it was demonstrated that in adolescents, false memory increased after multiple nights of experimentally reduced sleep opportunity, which is in line with a recent observation of higher tendency for false memory formation after 1 night of self‐reported short sleep duration in young adults (Frenda *et al*., 2014). The propensity to form false memories does not appear to be related to higher levels of subjective sleepiness, neither does it appear to be associated with a decline in vigilance. Decline in PVT performance was comparable after TSD and PSD in young adults, yet the effect size associated with false memory formation following TSD was greater than that after PSD. Furthermore, statistically, the number of lapses in the PVT did not account for sleep loss‐induced false memory formation uniformly in the two experiments.

In the present work, elevated false memory formation in sleep‐deprived individuals was likely a result of increased faulty encoding. While sleep loss can potentially perturb memory at both encoding and retrieval phases, the observation that poorer recognition can persist even after recovery sleep (Yoo *et al*., 2007) suggests it is weaker encoding that increases one's susceptibility to retroactive interference.

According to this account, veridical memory might be expected to be similarly affected by sleep loss. However, here, the effect of sleep loss on veridical memory may be masked by having veridical information presented again in the narrative phase of the misinformation paradigm. This would provide a second opportunity to encode information that, if source‐misattributed, could inflate veridical memory score. Indeed, when declarative materials are presented only once obviating re‐learning, sleep deprivation at encoding can result in poorer memory recognition (Yoo *et al*., 2007).

The comparable effect size of PSD on false memory formation in adolescents and young adults, despite the preservation of stage N3 sleep, challenges the notion that adolescents' memory can remain resilient to substantial sleep curtailment as long as the amount of slow wave sleep is not reduced (Voderholzer *et al*., 2011). Furthermore, in a sleep‐deprived state, adolescents are as vulnerable as young adults to the interfering effects of misleading post‐event information. More work in this area is warranted as many East Asian adolescents sleep 1–2 h less than their counterparts in Europe and Australia (Gradisar *et al*., 2011; Olds *et al*., 2010), but continue to excel in standardized measures of academic excellence (Organization for Economic Co‐operation and Development, 2014). The latter has led many to dismiss sleep curtailment as a negative factor weighing on memory and academic performance. Another potential avenue for research lies in investigating whether recovery sleep can reduce false memory formation. Prior work suggests that sleep‐related consolidation may preferentially benefit weaker memories (Drosopoulos *et al*., 2007), and can take place for several nights after encoding (Schonauer *et al*., 2015). This could potentially reduce memory impairment in persons sleep‐deprived at encoding. Another noteworthy point is that the misinformation paradigm was administered at different times of day in Experiments 1 and 2 (10:00 and 14:00 hours, respectively); yet, the effect size of PSD on the misinformation consistent response rate was similar, suggesting minimal circadian modulation of such effect from late morning to early afternoon.

In conclusion, multiple nights of restricted sleep result in an increase in false memory formation in adolescents, a group previously thought of as being resistant to cognitive impairment arising from sleep loss. In young adults, false memory formation is elevated after a night of TSD. Moreover, sleep history should be factored in when considering the veracity of eyewitness testimony.

## Author Contributions

J. C. Lo and M. W. L. Chee developed the study concept and design. Data were collected by J. C. Lo, S. Ganesan, P. L. H. Chong and R. L. F. Leong. J. C. Lo, S. Ganesan and P. L. H. Chong performed the data analysis and drafted the manuscript. M. W. L. Chee provided critical revisions. All authors approved the final version of the manuscript for submission.

## Conflict of Interest

The authors declare no conflict of interest.

## Supporting information


**Figure S1.** Relationship between misinformation consistent response rate and sleep.
