# Supplementary material for: Sleep deprivation increases formation of false memory
Source: J Sleep Res. 2016 Jul 5;25(6):673–82. doi: 10.1111/jsr.12436 (PMC5324644; doi:10.1111/jsr.12436)

**Figure S1.** Relationship between misinformation consistent response rate and sleep. Regression lines indicate the linear associations of misinformation consistent response rate with (A) total sleep time (TST), and the durations of (B) N1 sleep, (C) N2 sleep, (D) N3 sleep, (E) NREM sleep, and (F) REM sleep on the last manipulation night (M7; left panel) as well as the average across the first, fourth, and seventh manipulation nights (M1, M4, and M7; right panel). Data for the partial sleep deprivation (PSD) and the control groups were illustrated in gray circles and lines, as well as white circles and black lines respectively.


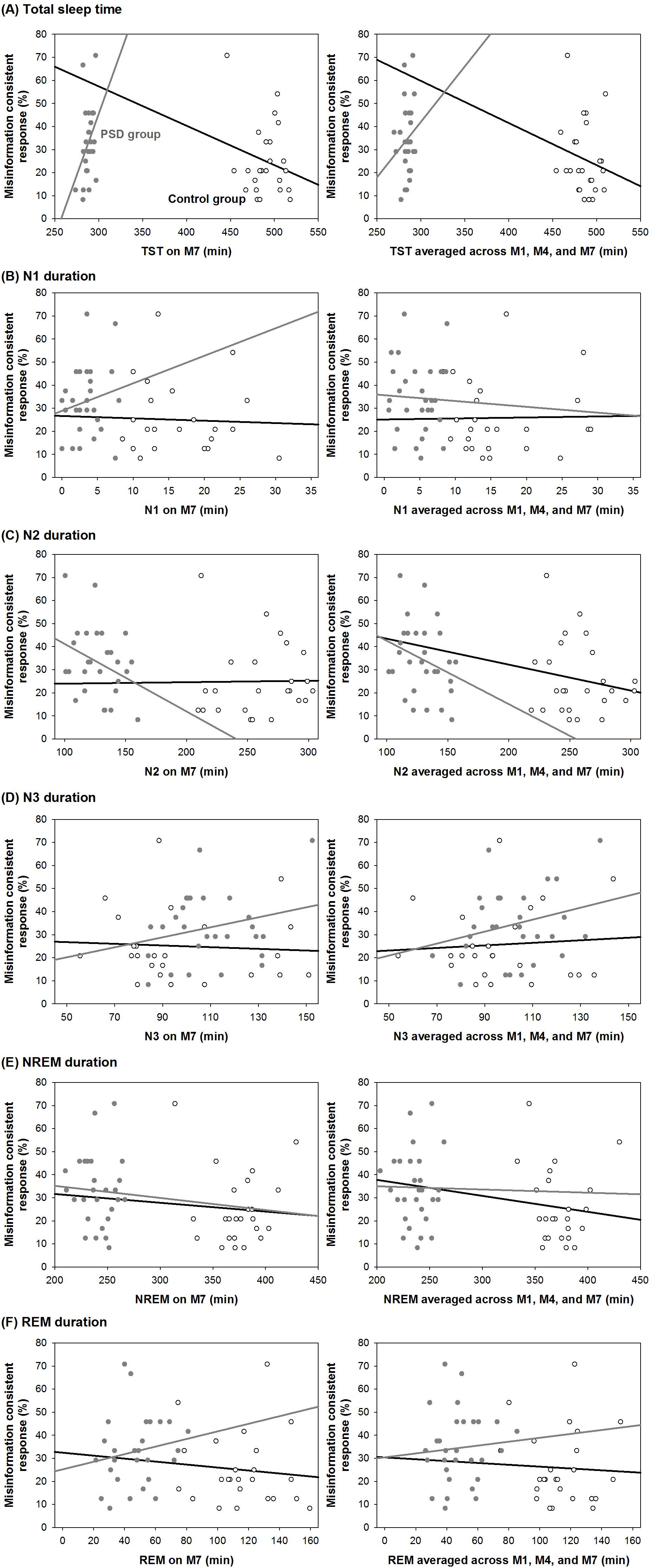


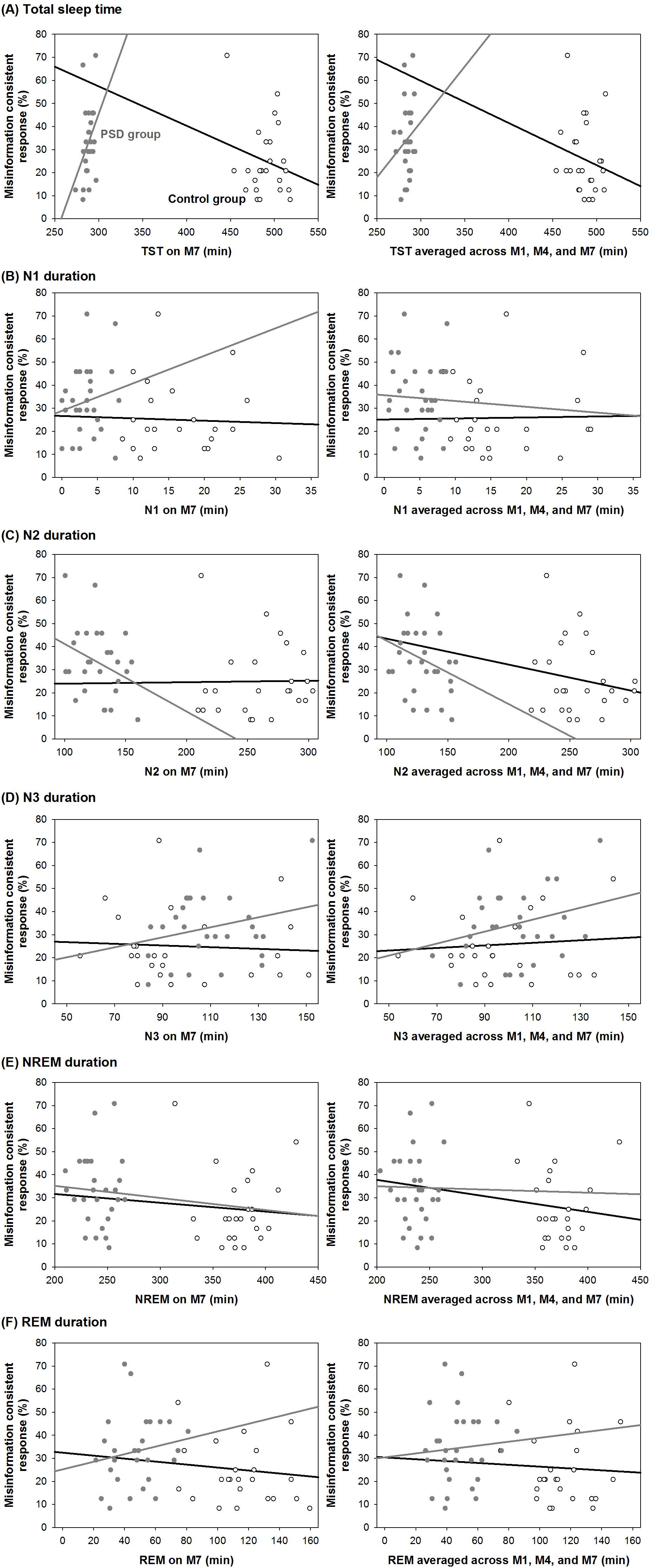

Supplement: Supplementary file 1 — Figure S1. Relationship between misinformation consistent response rate and sleep. [file JSR-25-673-s001.docx]
