# Supplementary material for: Sleep deprivation increases formation of false memory
Source: J Sleep Res. 2016 Jul 5;25(6):673–82. doi: 10.1111/jsr.12436 (PMC5324644; doi:10.1111/jsr.12436)
Supplement: Supplementary file 2 [file JSR-25-673-s002.pdf]

PEDIATRICS

Cognitive Performance, Sleepiness, and Mood in Partially Sleep Deprived Adolescents: The Need for Sleep Study

June C. Lo, PhD; Ju Lynn Ong, PhD; Ruth L.F. Leong, BSSc; Joshua J. Gooley, PhD; Michael W.L. Chee, MBBS

Centre for Cognitive Neuroscience, Neuroscience and Behavioral Disorders Program, Duke-NUS Medical School, Singapore

Study Objectives: To investigate the effects of sleep restriction (7 nights of 5 h time in bed [TIB]) on cognitive performance, subjective sleepiness, and mood in adolescents.

Methods: A parallel-group design was adopted in the Need for Sleep Study. Fifty-six healthy adolescents (25 males, age = 15–19 y) who studied in top high schools and were not habitual short sleepers were randomly assigned to Sleep Restriction (SR) or Control groups. Participants underwent a 2-w protocol consisting of 3 baseline nights (TIB = 9 h), 7 nights of sleep opportunity manipulation (TIB = 5 h for the SR and 9 h for the control groups), and 3 nights of recovery sleep (TIB = 9 h) at a boarding school. A cognitive test battery was administered three times each day.

Results: During the manipulation period, the SR group demonstrated incremental deterioration in sustained attention, working memory and executive function, increase in subjective sleepiness, and decrease in positive mood. Subjective sleepiness and sustained attention did not return to baseline levels even after 2 recovery nights. In contrast, the control group maintained baseline levels of cognitive performance, subjective sleepiness, and mood throughout the study. Incremental improvement in speed of processing, as a result of repeated testing and learning, was observed in the control group but was attenuated in the sleep-restricted participants, who, despite two recovery sleep episodes, continued to perform worse than the control participants.

Conclusions: A week of partial sleep deprivation impairs a wide range of cognitive functions, subjective alertness, and mood even in high-performing high school adolescents. Some measures do not recover fully even after 2 nights of recovery sleep.

Commentary: A commentary on this article appears in this issue on page 497.

Keywords: adolescents, cognitive performance, mood, partial sleep deprivation, sleepiness

Citation: Lo JC, Ong JL, Leong RL, Gooley JJ, Chee MW. Cognitive performance, sleepiness, and mood in partially sleep deprived adolescents: the need for sleep study. *SLEEP* 2016;39(3):687–698.

Significance

Some of the world's most sleep deprived students live in East Asia where students excel in standardized academic tests. This might reinforce the notion that 'mind over matter' can overcome negative effects of chronic sleep restriction. We found that in adolescents, partial sleep deprivation of comparable duration and severity to that examined in studies on young healthy adults elicited equivalent or greater neurobehavioral deficits across several cognitive domains. Residual effects on sustained attention, speed of processing, and subjective alertness can still be observed even after 2 nights of recovery sleep. That even students from top high schools are susceptible to neurobehavioral deficits should cause policymakers and parents to reconsider if sleep should continue to be sacrificed for the sake of academic achievement.

INTRODUCTION

Sleep curtailment in adolescents is a serious problem in many societies, but insufficient action is being taken to stem this tide. Approximately 75% of adolescents in the US[1] and more than 90% in Korea[2] and Japan[3] sleep less than the recommended 8–10 h a night.[4] Previously, the maturational delay in bedtime combined with early morning school were the principal reasons for shortened sleep in adolescence.[5] In recent years, increased electronic media use, higher homework load, and reduced parental control have contributed to further sleep curtailment in this age group.[6] In highly competitive societies in East Asia where voluntary sleep curtailment is most prevalent, there is widespread belief that greater effort and more time spent studying, perhaps at the expense of sleep, is mandatory for acceptable academic performance.[7] This viewpoint is sustained by the higher scores achieved on standardized tests by students from East Asian countries[8] who, on average, sleep 1 to 2 h less than their European[9,10] or Australian[10] counterparts. Although three decades of observational and experimental studies on sleep curtailment in adolescents have provided clear evidence for increased daytime sleepiness, the case for objective cognitive performance degradation following partial sleep deprivation has been less compelling,[6,11] prompting the current study.

Effects of Partial Sleep Deprivation on Subjective Sleepiness and Mood

Sleep restricted adolescents have been consistently found to be more sleepy. Results of observational studies have revealed shorter sleep duration to be associated with higher levels of subjective sleepiness.[12,13] Moreover, experimental studies have shown that just 1 night of 4- to 5-h sleep opportunity reduces sleep latency in the Multiple Sleep Latency Test[14–16] and increases levels of subjective sleepiness.[15] After 5 nights of 6.5 h of time in bed (TIB), higher levels of subjective sleepiness have been corroborated by parental assessment.[17]

Short sleep duration has also been associated with greater emotional lability.[18] Compared to a well-rested condition, 2 nights of sleep restriction lowered self-reported positive affect.[19] Elevated negative affect ratings were observed after 5 nights of restriction to 6.5 h of TIB for sleep.[20]

Cognitive Consequences of Partial Sleep Deprivation

In comparison to adults, the effects of shortened sleep on objectively measured cognitive performance in children and adolescents have been found to be relatively modest, leading some to suggest that adolescents may be more resistant to sleep loss.[21] Although several observational studies have found that speed of processing, sustained attention, working memory,

and executive function are poorer in children and adolescents who report shorter sleep,[22–24] other studies have failed to find a significant relationship between sleep duration and speed of processing,[23,25] working memory, or executive function.[12,23,25]

Experimental studies on the cognitive consequences of partial sleep deprivation in children and adolescents have yielded heterogeneous findings, possibly because of differences in the extent of partial sleep deprivation and the cognitive tasks used across studies. In relation to partial sleep deprivation, both the severity of sleep restriction each night and the number of nights sleep was restricted have generally been lesser than in adult studies. Most partial sleep deprivation studies in children and adolescents have either reduced TIB by only 1 h for a few nights[26] or have restricted sleep opportunity to 4 to 5 h for only 1 night.[14–16,27] Although partial sleep deprivation has been observed to impair attention,[26] working memory,[26] executive function,[16] and verbal creativity[16] in some studies, others have not found any significant decrement in attention,[14,15,27] executive function,[27] or speed of processing.[14,16,26]

Two studies investigated the cognitive effects of a longer period of sleep restriction. In one, 5 nights of sleep restricted to 6.5 h TIB resulted in increased student and parent reports of inattention, as well as problems with metacognition.[17] However, in a subset of these participants who underwent functional magnetic resonance imaging, the investigators found no objective deficit in working memory or executive function. These adolescent participants might have modulated task-related activation to mitigate any potentially deleterious effects of sleep restriction.[28]

In a second study,[21] participants restricted to 5, 6, 7, 8, or 9 h of TIB for 4 nights did not exhibit any deficit in attention, speed of processing, executive function, or working memory. Although total sleep time (TST) was reduced in each of the sleep-restricted groups, the duration of slow wave sleep was not affected, leading the investigators to propose that adolescents may be resilient to cognitive impairment following substantial sleep restriction because of the preservation of slow wave sleep.[21]

A recent meta-analysis[29] on both observational and experimental studies found that in school-age children, the correlation between short sleep duration and poor cognitive performance was very modest (r = 0.08). When various cognitive domains were analyzed separately, shorter sleep duration was only modestly associated with poorer executive function, and not at all with sustained attention – a cognitive domain highly sensitive to partial sleep deprivation in adults.[30,31]

In the current study, we evaluated the effect of 7 nights of partial sleep deprivation on adolescents, seeking to fill gaps left by previous studies. First, we recruited students from top high schools – the type of students many lay persons expect to transcend the need for sleep when motivated to attain desired goals. Second, the modest effects of partial sleep deprivation in prior experiments could have resulted from insufficiently severe sleep restriction compared to similar studies in adults. In addition, these milder degrees of sleep restrtriction are not representative of the sleep schedules encountered by students living in highly competitive societies. To examine this possibility, sleep was restricted to 5 h TIB for 7 consecutive nights. Third, to facilitate comparison with similar studies on adults, our test battery comprised tests commonly used in adults. An example is the Psychomotor Vigilance Task (PVT),[32] which is widely used in sleep deprivation studies on adults[33] but has not been used in studies on children and adolescents. Fourth, to enhance ecological validity of our findings, the current study was conducted in a dormitory instead of in a sleep laboratory. Although a natural setting was used, the instrumentation, tests, and test frequency were similar to those used in laboratory-based studies. In particular, sleep was evaluated using both actigraphy and polysomnography (PSG).

METHODS

Participants

Sixty participants were invited to participate in the Need for Sleep Study, a 2-w protocol aimed at characterizing changes in cognitive performance, subjective sleepiness, and mood associated with sleep curtailment in adolescents. Participants were between 15 and 19 y of age; had to have no history of any chronic medical condition, psychiatric illness, or sleep disorder; had a body mass index ≤ 30; were not habitual short sleepers (i.e. had an average actigraphically estimated TIB of < 6 h and no sign of sleep extension on weekends); had to consume fewer than five cups of caffeinated beverages a day; and must not have traveled across more than two time zones 1 mo prior to the experiment.

Participants were randomized into the sleep restriction (SR) and the control groups. They were not informed about their grouping until the first day of the 2-w protocol. Two participants withdrew several days prior to the study and one during the study for personal reasons. One participant did not comply with the experimental procedures and was excluded from all the analyses.

The resulting sample consisted of 56 participants (25 males, mean ± standard deviation of age = 16.6 ± 1.1 y). The SR (n = 30) and the control groups (n = 26) did not differ in age, sex distribution, body mass index, consumption of caffeinated beverages, nonverbal intelligence, levels of anxiety and depression, morningness-eveningness preference, levels of daytime sleepiness, symptoms of chronic sleep reduction, global score of the Pittsburgh Sleep Quality Index, and self-reported and actigraphically assessed sleep habits (Table 1; refer to the next section for details of screening instrumentation). Data from actigraphy during term time indicated that on weekdays, these participants slept less than the recommended 8–10 h,[4] and TIB and TST increased by more than 2 h from weekdays to weekends (Table 1).

Recruitment and Screening

This study was approved by the Institutional Review Board of the National University of Singapore. Participants were recruited through sleep education talks in two high-ranking high schools (see endnote A), advertisements on the laboratory and social networking websites, as well as by word of mouth. All interested participants and their legal guardians were invited to attend a briefing session. Written informed consent was obtained from each participant and a legal guardian.

The Pittsburth Sleep Quality Index[34] was used to assess self-reported sleep timing, duration, and quality, whereas the

Table 1—Characteristics for the sleep restriction and the control groups.

	Sleep Restriction Group		Control Group			
	Mean	SD	Mean	SD	t / χ^2	P
n	30	–	26	–	–	–
Age (y)	16.43	0.94	16.81	1.17	1.33	0.19
Sex (% males)	46.70	–	42.30	–	0.11	0.74
Body mass index	20.43	2.88	20.38	2.55	0.07	0.94
Caffeinated drinks per day	0.75	0.55	0.54	0.79	1.18	0.25
Raven's Advanced Progressive Matrices score	9.77	1.98	10.38	1.06	1.43	0.16
Beck Anxiety Inventory score	7.80	6.45	6.58	4.83	0.79	0.43
Beck Depression Inventory score	6.90	5.49	5.19	4.68	1.24	0.22
Morningness-Eveningness Questionnaire score	47.90	7.43	49.96	7.15	1.05	0.30
Epworth Sleepiness Scale score	7.77	3.59	6.19	3.57	1.64	0.11
Chronic Sleep Reduction Questionnaire						
Total score	34.50	5.77	33.81	5.13	0.47	0.64
Shortness of sleep	12.37	2.39	12.50	2.30	0.21	0.83
Irritation	6.97	1.85	6.77	1.58	0.43	0.67
Loss of energy	7.43	1.94	7.00	1.65	0.89	0.38
Sleepiness	7.73	1.66	7.54	1.75	0.43	0.67
Pittsburgh Sleep Quality Index						
TIB on weekdays (h)	6.12	1.03	5.94	1.14	0.63	0.54
TIB on weekends (h)	8.70	1.23	9.20	1.30	1.50	0.14
TIB on average (h)	6.86	0.87	6.87	0.87	0.07	0.95
TST on weekdays (h)	5.91	1.02	5.78	1.15	0.45	0.66
TST on weekends (h)	8.48	1.24	9.04	1.30	1.65	0.11
TST on average (h)	6.64	0.87	6.71	0.88	0.28	0.78
Global score	5.17	2.32	4.58	2.58	0.90	0.37
Actigraphy						
TIB on weekdays (h)	6.40	0.94	6.09	0.85	1.24	0.22
TIB on weekends (h)	8.46	1.08	8.45	1.25	0.99	0.99
TIB on average (h)	6.98	0.72	6.76	0.77	1.08	0.29
TST on weekdays (h)	5.61	0.86	5.37	0.73	1.11	0.27
TST on weekends (h)	7.46	1.10	7.53	1.14	0.21	0.84
TST on average (h)	6.14	0.66	5.99	0.62	0.89	0.38
Sleep efficiency (%)	87.86	5.46	88.45	4.66	0.42	0.68

SD, standard deviation; TIB, time in bed; TST, total sleep time.

Morningness-Eveningness Questionnaire[35] evaluated morningness-eveningness preference. Participants completed the Chronic Sleep Reduction Questionnaire[36] to evaluate symptoms of chronic sleep restriction, the Epworth Sleepiness Scale[37] to examine levels of daytime sleepiness, and the Berlin Questionnaire[38] to screen for obstructive sleep apnea. The Beck Anxiety Inventory[39] and the Beck Depression Inventory[40] were used to probe for anxiety and depression respectively. Nonverbal intelligence was assessed using the Raven's Advanced Progressive Matrices.[41] Participants wore an actiwatch (Actiwatch 2, Philips Respironics, Inc., Pittsburg, PA) for 1 w during term time to evaluate sleep patterns. They also filled in a sleep diary during that week, which provided additional information for identifying bedtime and wake time on the actogram.

Each participant who met the inclusion criteria was interviewed by JCL or RLL to ensure they would be comfortable interacting with other participants and research staff, as well as living away from home during the 2-w study period.

Two-Week Study Protocol

One week prior to the study, participants were required to adhere to a sleep-wake schedule that provided a 9-h nocturnal sleep opportunity (23:00–08:00). This was verified using wrist-worn actigraphy and was intended for circadian entrainment and for minimizing any effect of prior sleep restriction on sleep and cognitive performance.

The 2-w protocol (Figure 1A) was conducted in a boarding school after the school year had ended. In the first 3 nights (B1–B3), both SR and control participants had a 9 h nocturnal sleep opportunity (23:00–08:00) for adaptation and baseline characterization purposes. This was followed by a 7-night manipulation period (M1–M7) in which the SR group had 5 h (01:00–06:00) and the control group had 9 h (23:00–08:00) sleep opportunities. The protocol ended with 3 nights of 9-h recovery sleep (R1–R3: 23:00–08:00) for both groups.

All participants slept in twin-share, air-conditioned rooms, each with its own en-suite bathroom. Males and females were

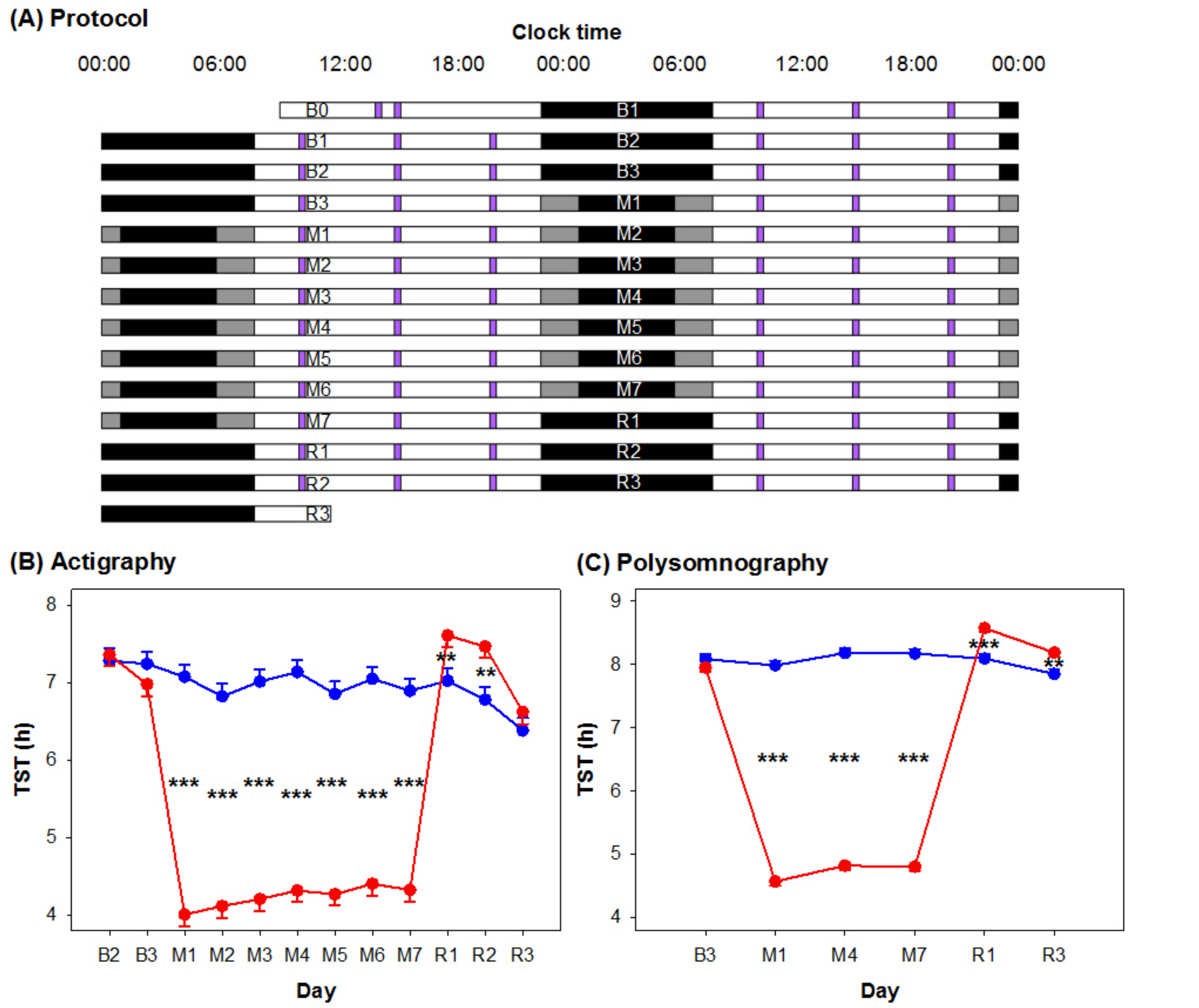

Figure 1—(A) Experimental protocol. The 2-w experimental protocol is illustrated in a double raster plot. Both the sleep restriction (SR) and the control groups had three adaptation and baseline nights (B1 to B3; time in bed [TIB] = 9 h), followed by 7 nights of sleep opportunity manipulation (M1 to M7; TIB = 5 h for SR [black bars] and 9 h for control [gray bars]), and 3 nights of recovery sleep (R1 to R3; TIB = 9 h). On most days, a cognitive performance test battery (purple bars) was administered at 10:00, 15:00, and 20:00. **(B)** Actigraphically and **(C)** polysomnographically assessed total sleep time (TST) of the SR (red lines) and the control (blue lines) groups from the adaptation and baseline period to the manipulation and recovery periods. Standard errors are illustrated. **P < 0.01; ***P < 0.001 for group contrasts.

housed in different buildings. The SR and the control groups were housed on different floors. Windows in each bedroom were fitted with blackout panels to prevent participants from being woken up by sunlight. Participants were provided with earplugs and allowed to adjust room temperature according to their own comfort. Apart from scheduled sleep periods, meal times, and cognitive testing periods, participants spent most of their time in a common room that received natural as well as artificial lighting. Participants were allowed to play board games, read, study, watch movies, and play games on their own electronic devices, in addition to interacting with research staff and other participants. Participants were under constant supervision of the research staff. Three main meals were served each day, and snacks were provided upon request. Caffeinated drinks, napping, and strenuous physical exercise were prohibited.

Sleep-wake patterns were continuously assessed with wrist-worn actigraphy, except for the first night, i.e. night B1, when all the actiwatches were charged. Each day, a computerized cognitive performance test battery was administered at 10:00, 15:00, and 20:00 (except for the first day [i.e. day B0]; Figure 1; see endnote B). Polysomnographic recordings were obtained on 7 nights: B1 and B3 for adaptation and baseline assessment, M1, M4, and M7 to monitor sleep changes from the beginning to the end of the manipulation period, and R1 and R3 for characterizing recovery sleep. Pulse oximetry was used on the first night to evaluate oxygen desaturations that might indicate undiagnosed obstructive sleep apnea. Here, we will report

findings regarding TST, whereas the sleep macrostructure and microstructure findings will be published separately.

Cognitive Performance Test Battery

A computerized cognitive performance test battery was administered on 57 identical laptop computers (Acer Aspire E11, Acer Inc, Taipei, Taiwan). All tests were programmed in E-Prime 2.0 (Psychology Software Tools, Inc., Sharpsburg, PA). Each test battery lasted for approximately 25 min. Participants were required to wear earphones throughout the test battery to minimize distractions and for tone presentation during certain tasks. The test battery comprised 7 tasks presented in the following order: the Karolinska Sleepiness Scale (KSS)[42] the Sustained Attention to Response Task (SART),[43] the Symbol Digit Modalities Test (SDMT),[44] the verbal 1 and 3-back tasks,[31] the Mental Arithmetic Test (MAT),[45] the Positive and Negative Affect Scale (PANAS),[46] and the PVT.[32]

In the KSS,[42] participants rated their current level of subjective sleepiness using a nine-point Likert scale (1 very alert, 9 very sleepy, great effort to keep awake).

The SART[43] was used to assess sustained attention. Numbers ranging from 0 to 9 were presented sequentially on the screen for 250 msec, and participants were required to respond by pressing the spacebar on every trial, except when the target number '8' appeared. The target to non-target ratio was 15:85, and the inter-stimulus interval was fixed at 900 msec. Two nonparametric measures of sensitivity (A') and response bias (B''_D) were used to quantify performance. A' is a measure of a participant's ability to discriminate between targets and non-targets, and is computed using the hit rate (number of non-target trials responded to × 100/85) and false alarm rate (number of target trials responded to × 100/15). A' ranges from 0 to 1, with 0.5 indicating performance at chance level. B''_D is a measure of a participant's tendency toward liberal ($B''_D < 0$) or conservative ($B''_D > 0$) response behavior, where the former favors more responses and so is more likely to lead to responses when they are not required; the latter favors withholding responses, and as a result is less likely to result in false alarms when responses are not required. Neutrality is centered at 0 ($B''_D = 0$).

The two measures were derived with the following formula.[47,48]

$$\text{For } hit > fa,\ A' = \frac{1}{2} + \frac{(hit - fa) \times (1 + hit - fa)}{4 \times hit \times (1 - fa)}$$

$$\text{For } fa > hit,\ A' = \frac{1}{2} + \frac{(fa - hit) \times (1 + fa - hit)}{4 \times fa \times (1 - hit)}$$

$$B''_D = \frac{(1 - hit) \times (1 - fa) - (hit \times fa)}{(1 - hit) \times (1 - fa) + (hit \times fa)}$$

The SDMT[44] was used to measure speed of processing. In this task, participants were shown a key displaying nine pairs of digits and symbols. On every trial, a symbol appeared below the key, and participants were required to respond by inputting its corresponding digit (ranging from 1 to 9 on the keyboard) as quickly as possible. If participants did not respond in 15 sec, a beeping tone was presented until a response was recorded. This task lasted for 2 min. The total number of correct trials was used as the critical measure.

Verbal n-back tasks[31] were used to assess working memory and executive function. In this task, alphabets were presented sequentially for 1,000 msec with 3,000 msec inter-stimulus interval. Participants were required to decide whether the current stimulus matched with the one shown one (1-back) or three (3-back) items ago. The match to mismatch ratio was 8:24. We used the formulas stated above to derive measures of sensitivity (A') and response bias (B''_D) to quantify performance.

The MAT[45] was used to measure speed of processing. This took the form of addition problems involving pairs of two-digit numbers that were shown on screen, and participants were required to solve them as quickly as possible. A beeping tone was presented if participants did not respond within 15 sec. The total number of correct trials in this 4-min task was used as the critical measure.

The PANAS[46] was used to assess positive and negative affect. Participants were shown 20 adjectives with 10 describing positive mood and 10 describing negative mood. Participants needed to respond using a five-point Likert scale (1 very slightly, 5 extremely).

A 10-min PVT[32] was used to measure levels of sustained attention. At random intervals varying from 2,000 msec to 10,000 msec, a counter on the computer screen started counting, and participants were required to respond as quickly as possible by pressing a key. A beeping tone was presented if no response was detected 10,000 msec after stimulus onset. The number of lapses (responses exceeding 500 msec) recorded during each PVT test was used as a measure of sustained attention.

Actigraphy

Participants wore an actiwatch (Actiwatch 2, Philips Respironics, Inc., Pittsburgh, PA) around the wrist of their non-dominant hand during term time for screening purposes, during the 1-w pre-study period for verifying their compliance with the specified sleep schedule, as well as during the 2-w protocol. Data were collected at 2 min resolution and were scored with the Actiware software (version 6.0.2). TST was calculated using a medium sensitivity algorithm, with which an activity count greater than or equal to 40 was defined as waking. Participants also kept a sleep diary during the actigraphically monitored periods at home.

Polysomnography

Electroencephalography (EEG) was performed using a SOMNOtouch recorder (SOMNOmedics GmbH, Randersacker, Germany) from two channels (C3 and C4 in the international 10–20 system) referenced to the contralateral mastoids. The common ground and reference electrodes were placed at Cz and FPz. Electrooculography (EOG) and submental electromyography (EMG) were also used. Impedance was kept below 5 kΩ for EEG electrodes and below 10 kΩ for EOG and EMG electrodes. Signals were sampled at 256 Hz and filtered between 0.2 and 35 Hz for EEG and between 0.2 and 10 Hz for EOG.

Sleep scoring analyses were performed using the FASST toolbox (http://www.montefiore.ulg.ac.be/~phillips/FASST.html).

EEG signals were band-pass filtered between 0.2 and 25 Hz. Scoring was performed visually by trained technicians following the criteria set by the American Academy of Sleep Medicine Manual for the Scoring of Sleep and Associated Events.[49]

Statistical Analyses

Statistical analyses were performed with SAS 9.3 (SAS Institute, Cary, NC). We used a general linear mixed model with PROC MIXED to determine the effects of group, day (from day B3 to R2), and the group × day interaction on cognitive performance, sleepiness, and mood averaged across the three test batteries each day. We included performance on day B2 (see endnote C) as a covariate to control for group differences in baseline performance. To quantify the local effect size of partial sleep deprivation on each measure, we used a similar statistical model but excluded the recovery days to compute Cohen f^2 of the group × day interaction.[50] The cutoffs for small, medium, and large effect sizes were 0.02, 0.15, and 0.35, respectively.[51] We excluded data from the first five test batteries on days B0 and B1 in all the analyses to minimize influence of practice effects.

To assess the efficacy of our manipulation of sleep opportunities, we also used a general linear mixed model to determine the effects of group, day (from night B2 to R3 for actigraphic data, and from night B3 to R3 for PSG data), and group × day interaction on TST. PSG data from night B1, i.e., the adaptation night, was not included in the analysis. To ensure that the two groups followed the 9-h sleep schedule and did not differ in sleep duration the week prior to the 2-w protocol, we performed independent-samples *t* tests on actigraphically estimated TIB and TST.

RESULTS

Sleep Duration before and during the Protocol

One week before the 2-w protocol, both groups complied with the 9-h sleep schedule at home (mean ± standard error of the mean of TIB of the SR group: 8.78 ± 0.07 h versus control group: 8.84 ± 0.04 h, $t(53) = 0.68$, $P = 0.50$). There was no significant group difference in actigraphically estimated TST (SR: 6.89 ± 0.16 h versus control: 6.94 ± 0.11 h, $t(53) = 0.25$, $P = 0.80$), suggesting that sleep history did not differ between the two groups. The actigraphically estimated TST of 6.9 h appears short but is readily explained by actigraphy *underestimating* TST by approximately 1 h relative to PSG (see next section). As such, it is likely that our participants were well rested prior to the study.

We found that (1) the SR and the control groups had similar TST at baseline, (2) the partial sleep deprivation manipulation resulted in a large reduction in daily TST, and (3) the SR group had greater TST during the recovery nights. In the ensuing material, we provide a detailed breakdown of these points.

Actigraphy during the 2-w protocol revealed a significant group × day interaction on TST ($F(11,466) = 54.58$, $P < 0.001$). The two groups had similar actigraphically estimated TST on baseline nights (e.g., on B3, SR: 6.98 ± 0.15 h versus control: 7.24 ± 0.16 h, P = 0.23). During the manipulation period, TST was reduced to 4.01–4.41 h in the SR group and remained at approximately 7 h for the control group (Figure 1B). On the first 2 recovery nights, the SR group slept for 7.61 ± 0.15 h and 7.46 ± 0.15 h respectively, both longer than the last baseline night (P < 0.001 and P = 0.008) and significantly longer than the control group (R1: 7.02 ± 0.16 h, P = 0.01; R2: 6.78 ± 0.16 h, P = 0.002). On the third recovery night, TST of the SR group approached baseline level (P = 0.07), and the group difference disappeared (6.62 ± 0.16 h versus 6.38 ± 0.16 h, P = 0.23).

Actigraphy *underestimated* sleep duration by approximately 1 h relative to PSG. This systematic bias was less with higher sleep efficiency (i.e., as TST approached TIB) (Figure S1, supplemental material), and this was independent of the duration of sleep opportunity (TIB). Nevertheless, polysomnographic assessment of TST in response to sleep curtailment was, in general, congruent with the actigraphy findings (Figure 1C). The group × day interaction was statistically significant on TST ($F(5,179) = 572.14$, $P < 0.001$). TST in the last baseline night did not significantly differ between the two groups (SR: 7.95 ± 0.07 h versus control: 8.09 ± 0.07 h, P = 0.16). TST was maintained between 7.99 h and 8.18 h for the control group. The SR group showed a significant increase in TST from the beginning to the middle of the manipulation period (4.57 ± 0.07 h and 4.82 ± 0.07 h, P = 0.001). This was then maintained until the end of the sleep opportunity manipulation period (4.81 ± 0.07 h). In the first recovery night, not only was the SR group's TST significantly longer than the control group's (8.58 ± 0.07 h versus 8.09 ± 0.07 h, P < 0.001), it was significantly elevated from the baseline level (P < 0.001). On the third recovery night, TST of the SR group remained above baseline (P = 0.004) and significantly longer than the control (8.19 ± 0.07 versus 7.85 ± 0.08 h, P = 0.001).

Effects of Partial Sleep Deprivation on Subjective Sleepiness, Cognitive Performance, and Mood

Cognitive performance, subjective sleepiness, and mood in the SR group were affected by partial sleep deprivation, as evidenced by a decrement in performance or reduced rate of improvement. Two nights of recovery sleep were insufficient to return performance to baseline levels on measures of sustained attention and subjective sleepiness (Figure 2). Although recovery sleep might have restored performance improvement in speed of processing tasks, performance of individuals with prior sleep restriction remained poorer than the well-rested control group. In general, the control group showed relatively stable cognitive performance, levels of subjective sleepiness, and mood throughout the protocol.

In evaluating sustained attention, we found a group × day interaction on the number of lapses in the PVT ($F(9,456) = 9.09$, $P < 0.001$). The SR group showed a monotonic increase in the number of lapses throughout the partial sleep deprivation period. The number of lapses was significantly reduced after the first recovery sleep episode (P < 0.001), but remained elevated relative to baseline after the first two nights of recovery sleep (P < 0.001; left panel in Figure 2A). Performance on the SART was less affected by partial sleep deprivation. Although the group × day interaction was also significant for *A'* in the SART ($F(9,457) = 2.02$, $P = 0.04$), a noticeable decrease in *A'* was found only after 4 nights of partial sleep deprivation.

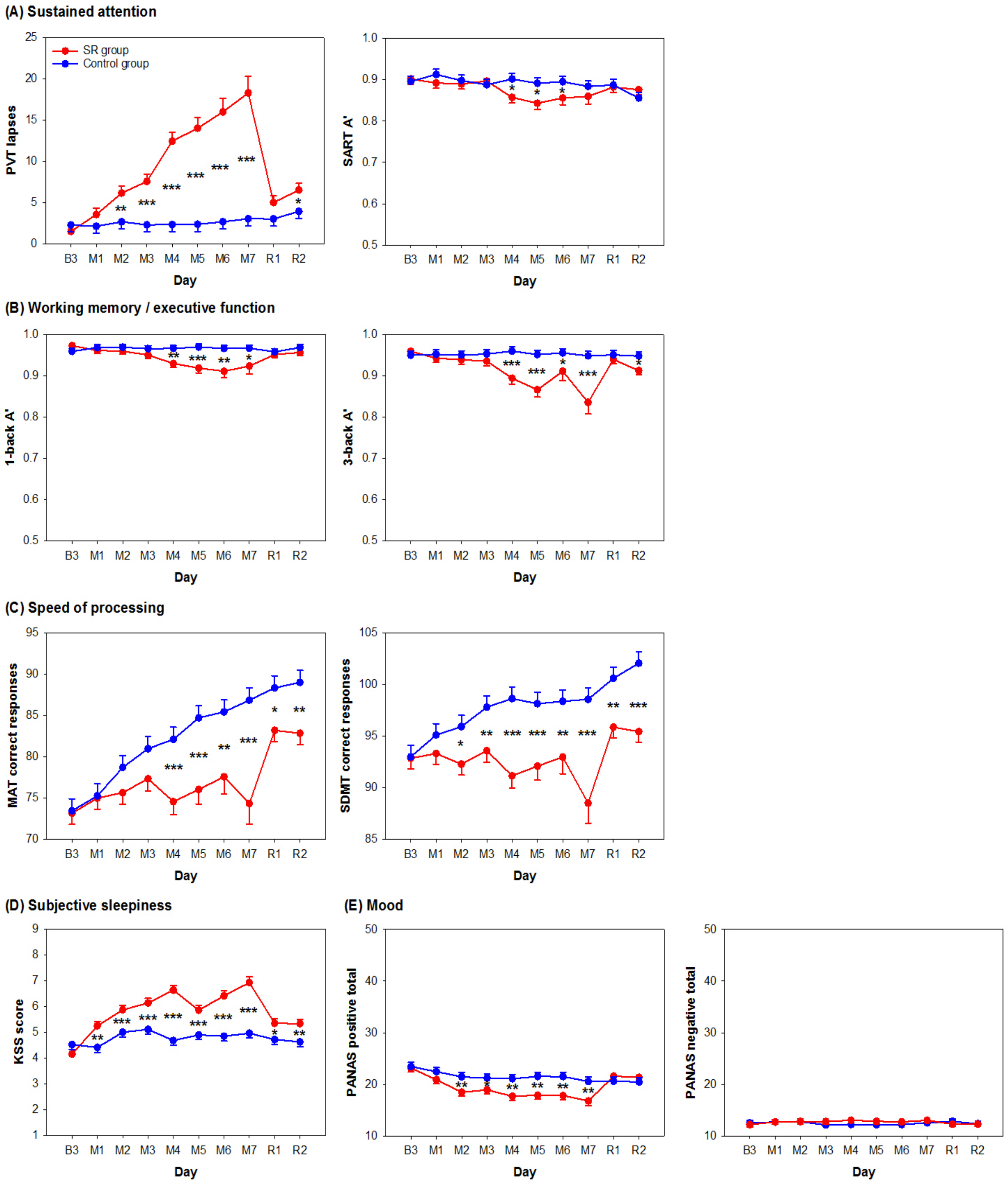

Figure 2—Effects of partial sleep deprivation on cognitive performance, subjective sleepiness, and mood. Daily average and standard errors of the sleep restriction (SR) (red lines) and the control (blue lines) groups from the days after the last baseline night (B3), after 1 to 7 nights of sleep manipulation (M1 to M7), and after 2 nights of recovery sleep (R1 and R2) were plotted for **(A)** sustained attention as indicated by the number of lapses in the Psychomotor Vigilance Task (PVT) and the sensitivity measure (*A'*) in the Sustained Attention to Response Task (SART), **(B)** working memory and executive functions as indicated by *A'* in the verbal 1- and 3-back tasks, **(C)** speed of processing as indicated by the number of correct responses in the Mental Arithmetic Test (MAT) and the Symbol Digit Modalities Test (SDMT), **(D)** subjective sleepiness level as indicated by score on the Karolinska Sleepiness Scale (KSS), and **(E)** positive and negative mood as indicated by the score on the Positive and Negative Affect Scale (PANAS). *P < 0.05; **P < 0.01; ***P < 0.001 for group contrasts.

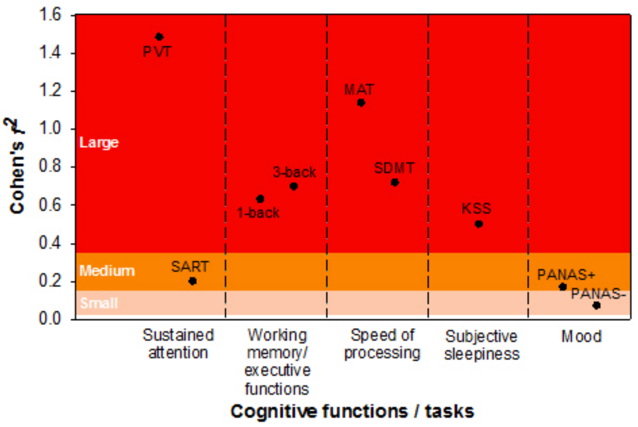

Figure 3—Effect size of partial sleep deprivation on cognitive performance, subjective sleepiness, and mood. Effect size is indicated by the local effect size (Cohen f^2) of group × day interaction on each cognitive measure (refer to the Methods section for further details). KSS, Karolinska Sleepiness Scale; MAT, Mental Arithmetic Test; PANAS, Positive and Negative Affect Scale (+, score on the positive affect subscale; −, score on the negative affect subscale); PVT, Psychomotor Vigilance Task; SART, Sustained Attention to Response Task; SDMT, Symbol Digit Modalities Test.

Performance returned to baseline levels after only 1 night of recovery sleep (P = 0.17; right panel in Figure 2A). This decrement in discriminability between targets and non-targets could not be explained by changes in response bias as B''_D was not affected at all by partial sleep deprivation (group × day interaction: $F(9,457) = 1.30$, P = 0.23; Figure S2A, supplemental material). In terms of effect size, performance in the PVT was the most sensitive to partial sleep deprivation of all the tests in this study ($f^2 = 1.48$; Figure 3). In comparison, the A' in SART, was much less affected by sleep restriction ($f^2 = 0.20$; Figure 3).

In terms of working memory and executive function, we observed a significant group × day interaction on A' in the verbal 1-back task ($F(9,456) = 2.45$, P = 0.01). A' declined after 4 nights of sleep restriction and returned to baseline levels after one recovery sleep episode (P = 0.06; left panel of Figure 2B). The group × day interaction on B''_D in the verbal 1-back task was not statistically significant ($F(9,457) = 1.70$, P = 0.09), and no significant group difference in the tendency toward conservative response behavior was observed throughout the protocol (left panel of Figure S2A). Hence, the decrement in A' in the SR group could not be accounted for by response bias. In the verbal 3-back task, there was also a significant group × day interaction on A' ($F(9,457) = 3.20$, P < 0.001). A' decreased after 4 nights of partial sleep deprivation and returned to baseline level after 1 night of recovery sleep (P = 0.15; right panel of Figure 2B) as observed in the verbal 1-back task. B''_D in the verbal 3-back task did not reveal a statistically significant group × day interaction ($F(9,457) = 1.51$, P = 0.14; right panel of Figure S2B) and hence, could not explain the decrease in A' induced by sleep loss. The size of the partial sleep deprivation effect on 1- and 3-back was similar in magnitude ($f^2 = 0.63$ and 0.70; Figure 3), suggesting that cognitive decrement induced by partial sleep deprivation did not change with executive load.

In both the MAT and the SDMT, which are tests of speed of processing, performance improved with repeated testing, but this was attenuated in the SR group relative to the control group (group × day interaction for the MAT: $F(9,456) = 4.33$, P < 0.001; for the SDMT: $F(9,456) = 4.02$, P < 0.001). Interestingly, the largest improvement in both tasks was demonstrated by the SR group across the first recovery night (P < 0.001 for both tasks; Figure 2D). Nevertheless, after 2 nights of recovery sleep, performance of the SR group remained significantly poorer than the control group (P < 0.003 for both tasks). Although both speed of processing tasks revealed a similar pattern, performance in the MAT was a more sensitive measure of sleep loss than the SDMT ($f^2 = 1.14$ and 0.72; Figure 3).

Subjective sleepiness evaluated using the KSS showed a significant group × day interaction ($F(9,457) = 9.32$, P < 0.001). KSS score was elevated after only 1 night of partial sleep deprivation (P < 0.001), progressively increased thereafter, and plateaued toward the end of the manipulation period. Although KSS score in the SR group decreased after 1 night of recovery sleep (P < 0.001), it was still higher than the baseline value (P < 0.001) and at the level observed after 1 night of partial sleep deprivation (P = 0.61). This remained so even after the second recovery night (versus baseline: P < 0.001; versus M1: P = 0.65; Figure 2D). The effect of partial sleep deprivation on subjective sleepiness was in the medium range ($f^2 = 0.50$; Figure 3).

We found a significant group × day interaction on positive mood ($F(9,456) = 4.71$, P < 0.001). Positive mood decreased progressively during partial sleep deprivation, leveled off toward the end of the manipulation period, and returned to the baseline level after 1 night of recovery sleep (P = 0.36; left panel of Figure 2E). In contrast, the group × day interaction on negative mood was not statistically significant ($F(9,457) = 1.15$, P = 0.33). Negative mood appeared to stay at a low level throughout the protocol for both the SR and the control groups (main effect of day: $F(9,457) = 0.61$, P = 0.79; right panel of Figure 2E). Effect size measures showed that partial sleep deprivation had a medium effect on positive affect ($f^2 = 0.17$), but only a small effect on negative affect ($f^2 = 0.07$; Figure 3).

DISCUSSION

Restricting adolescents' sleep to 5 h TIB for 7 nights led to cumulative degradation of sustained attention, working memory, executive function, and speed of processing. In contrast, the increase in subjective sleepiness and the reduction in positive mood leveled off in the course of the experiment. Residual effects on sustained attention and subjective sleepiness persisted after 2 nights of 9 h recovery sleep opportunity. Adolescents in the control condition consistently slept approximately 8 h each night, maintained their baseline levels of cognitive performance, subjective sleepiness, and mood, and even demonstrated improvement in speed of processing.

Partial Sleep Deprivation Affects Even Academically Strong Students

Perhaps the most important finding of the current work is that even students from top schools who regularly sleep 2 to 3 h less than recommended for their age on weekday nights

experience significant neurobehavioral deficits when exposed to partial sleep deprivation.

Prior studies on adolescents with perhaps one exception[21] have used less harsh sleep restriction than that used here. Experimental studies of partial sleep deprivation in adults have used 3 to 6 h TIB for a minimum of 7 nights.[31,52,53] This constitutes about 2 to 4 h less actual sleep a night, assuming a norm of 7 to 8 h.[54] For adolescents, studies using 5 h TIB are in theory comparable to adult studies. A meta-analysis summarizing sleep duration data in the past century has shown that sleep in children and adolescents has decreased by about 75 min from the 20th century, with Asia showing one of the fastest rates of reduction.[55] This perhaps can be due to a disproportionately larger amount of time spent on school work in East Asia than in Western developed countries.[56] A survey in Korea involving a nationally representative sample of over 130,000 adolescents found that 43% reported sleeping less than 6 h each night.[2] Preliminary data from students of one of the feeder schools to the current work showed that on average, the actigraphically estimated TST was below 5.5 h during weekdays (unpublished data). As such, the severity and duration of sleep restriction used here has real-world relevance.

The severity of neurobehavioral deficits we observed in adolescents is comparable to if not greater than that observed with adults exposed to a similar degree of partial sleep deprivation. For example, young adults showed about 10 lapses in the PVT after 7 nights of 4 h TIB,[53] whereas an average of 18 lapses were found after 7 nights of 5 h TIB in the current study. Although many students seek to emulate elite performers who sleep little, the current data show that even students from the top performing country in a global test on reading, mathematics and science[8] are not spared and experience significant neurobehavioral deficits when undergoing partial sleep deprivation.

Sustained Attention is the Most Affected Cognitive Domain, as in Adults

Previous studies have suggested that executive function and not attention is the cognitive domain most affected in partially sleep deprived children and adolescents.[11,16,57] These prior studies may not have found strong effects on attention because of differences in tests used to measure attention. The PVT is the most widely used test of sustained attention in adults. We detected monotonic deterioration in vigilance over the 7 nights of sleep restriction. The SART was less sensitive ($f^2 = 0.20$) than the PVT ($f^2 = 1.48$; Figure 3) highlighting the differential sensitivity to multi-night sleep restriction across tasks evaluating the same cognitive domain.

In terms of effect size, decline in speed of processing was the next most affected cognitive domain. This was slightly surprising given the absence of significant effects on this domain in prior studies on partial sleep deprivation in children and adolescents.[14,16,21,26] As in the case of sustained attention, we speculate that difference in task selection and severity of sleep restriction could explain these discrepancies. Although 1 night of recovery sleep might have restored the learning ability of the SR group, their performance failed to catch up with that of the control group. Whether additional nights of sufficient sleep can eliminate this group difference in performance remains to be investigated.

Working memory and executive function evaluated with 1- and 3-back tests were significantly affected by sleep restriction with an effect size of about half of that observed with the PVT. Interestingly, there was no additional decline in performance with increasing executive load (3 back versus 1 back). The absence of an incremental effect of load is similar to that observed with adults undergoing partial sleep deprivation[31] as well as visual short term memory and total sleep deprivation[58–60] and suggests that perceptual and attentional degradation[61] independently or together with maintenance failure[62] could underlie the performance decline attributed to executive function in the sleep deprived state.

Two Nights of Recovery Sleep may not be Enough but Cognitive Domain Matters

Two nights of recovery sleep may not be sufficient to achieve a complete recovery in sustained attention, speed of processing, and alertness after 1 w of relatively severe sleep restriction in adolescents. These findings are reminiscent of a study where healthy young adults were restricted to 4 h TIB for 5 nights and the duration of recovery sleep was varied. Even 10 h of recovery sleep for a single night was insufficient to completely restore sustained attention to baseline levels, although speed of processing was restored.[63]

Particularly relevant to hard driving students, the residual effects of sleep deprivation may cumulate and subsequent exposure to sleep restriction following incomplete recovery may result in disproportional decline in performance (Dinges, unpublished data). Relevant to this point, the relative plateauing of subjective sleepiness compared to monotonically declining sustained attention and reduced improvement in speed of processing[53,64] could cause adolescents to underestimate the extent of their objective neurobehavioral deficit. Of particular concern in societies where sacrificing sleep for academic success is prevalent is that chronic fatigue becomes a new societal norm.[65]

Poorer Positive Mood with Sleep Restriction

A decline in positive mood was observed after 2 nights of sleep restriction, similar to one previous study.[19] However, unlike another study,[20] we did not find an effect on negative mood. Although negative mood appeared to remain unaffected, many students remarked that the test items, e.g., guilty, scared, and afraid, were irrelevant to them. Sleep has been shown to modulate the processing of emotional memory.[66–68] Although this may have survival value, it could have negative effects on mental health. Indeed, a large behavioral risk factor survey found that shorter self-reported sleep duration in adolescents was associated with higher likelihood of reporting depressive symptoms and suicidal ideation.[2]

Differences in Adolescent Sleep Assessed by Wrist Actigraphy and PSG

The Bland-Altman plot (Figure S1) indicates that when sleep efficiency was high (i.e., when TST approached TIB), there was overall good concordance between sleep duration measured by both actigraphy and PSG, but when sleep efficiency was low, there was a systematic *underestimation* of TST for actigraphy. The underlying reasons for the underestimation, rather

than overestimation,[69] of TST by actigraphy in our sample of adolescents and for the increased discrepancy between sleep duration assessed by PSG and wrist actigraphy as a function of sleep efficiency are unknown and remain to be investigated.

Limitations and Future Studies

Sleep restriction was achieved by delaying bedtime and advancing wake time by 2 h so that the midpoints were aligned for both the sleep periods and the wake periods throughout the protocol to minimize circadian phase shifting. However, because test batteries were run at the same clock times, the duration of preceding wakefulness was always 2 h longer for the SR group during the manipulation period. The cognitive decrement associated with partial sleep deprivation might thus be accentuated by a longer duration of prior wakefulness before testing.

Our 7-night sleep restriction period was longer than the typical 5 study nights of 1 w when students curtailed their sleep. Although this might potentially limit the generalizability of our findings, it is not uncommon for highly competitive students to continue sleeping less than recommended on weekends in order to study. Furthermore, our finding that some cognitive functions failed to return to baseline levels after 2 nights of recovery sleep strongly signal the need to systematically evaluate the long-term effects of repeated cycles of sleep restriction and recovery on neurobehavioral deficits. Although we have unequivocally demonstrated neurobehavioral deficits using a standardized cognitive battery, the effect on ability to learn, to retain information, and to creatively reorganize learned material was not assessed. These higher-order cognitive functions are of critical interest and remain to be evaluated in future studies.

CONCLUSION

Partial sleep deprivation in adolescents of comparable duration and severity to that examined in studies on young healthy adults elicited equivalent or greater neurobehavioral deficits across several cognitive domains. Residual effects on sustained attention, speed of processing, and subjective alertness can still be observed even after 2 nights of recovery sleep. That even students from top high schools are susceptible to neurobehavioral deficits following partial sleep deprivation should cause policymakers and parents to reconsider if sleep should continue to be sacrificed for the sake of academic achievement.

ENDNOTES

A: Singapore was the top-ranked country out of 65 countries in the 2012 PISA examinations.[8] Most of our participants came from top ranked schools. All participants stayed in the boarding school during the 2-w protocol.

B: The label of day indicates the wake period after the corresponding sleep period. For example, day B2 refers to the day after the second baseline night, but before the third baseline night. This highlights the effect of sleep history on subsequent cognitive performance.

C: Performance on day B3 was not used as a covariate because these data were included in the effect of day in the statistical model. This model allows the evolution of cognitive performance, subjective sleepiness, and mood from the last baseline day (day B3) to be depicted.

REFERENCES

1. National Sleep Foundation. Sleep in American poll: Teens and Sleep. Washington, DC: National Sleep Foundation, 2006.
2. Do YK, Shin E, Bautista MA, Foo K. The associations between self-reported sleep duration and adolescent health outcomes: what is the role of time spent on Internet use? Sleep Med 2013;14:195–200.
3. Ohida T, Osaki Y, Doi Y, et al. An epidemiologic study of self-reported sleep problems among Japanese adolescents. Sleep 2004;27:978–85.
4. Hirshkowitz M, Whiton K, Albert SM, et al. National Sleep Foundation's sleep time duration recommendations: methodology and results summary. Sleep Health 2015;1:40–3.
5. Carskadon MA. Sleep in adolescents: the perfect storm. Pediatr Clin North Am 2011;58:637–47.
6. Bryant NB, Gomez RL. The teen sleep loss epidemic: what can be done? Trans Issues Psychol Sci 2015;1:116–25.
7. Hsin A, Xie Y. Explaining Asian Americans' academic advantage over whites. Proc Natl Acad Sci U S A 2014;111:8416–21.
8. Organization for Economic Co-operation and Development. PISA 2012 results in focus: what 15-year-olds know and what they can do with what they know. 2014. Available from: http://www.oecd.org/pisa/keyfindings/pisa-2012-results-overview.pdf.
9. Gradisar M, Gardner G, Dohnt H. Recent worldwide sleep patterns and problems during adolescence: a review and meta-analysis of age, region, and sleep. Sleep Med 2011;12:110–8.
10. Olds T, Blunden S, Petkov J, Forchino F. The relationships between sex, age, geography and time in bed in adolescents a meta-analysis of data from 23 countries. Sleep Med Rev 2010;14:371–8.
11. Kopasz M, Loessl B, Hornyak M, et al. Sleep and memory in healthy children and adolescents - a critical review. Sleep Med Rev 2010;14:167–77.
12. Anderson B, Storfer-Isser A, Taylor HG, Rosen CL, Redline S. Associations of executive function with sleepiness and sleep duration in adolescents. Pediatrics 2009;123:e701–7.
13. Wolfson AR, Carskadon MA. Sleep schedules and daytime functioning in adolescents. Child Dev 1998;69:875–87.
14. Carskadon MA, Harvey K, Dement WC. Acute restriction of nocturnal sleep in children. Percept Mot Skills 1981;53:103–12.
15. Fallone G, Acebo C, Arnedt JT, Seifer R, Carskadon MA. Effects of acute sleep restriction on behavior, sustained attention, and response inhibition in children. Percept Mot Skills 2001;93:213–29.
16. Randazzo AC, Muehlbach MJ, Schweitzer PK, Walsh JK. Cognitive function following acute sleep restriction in children ages 10-14. Sleep 1998;21:861–8.
17. Beebe DW, Fallone G, Godiwala N, et al. Feasibility and behavioral effects of an at-home multi-night sleep restriction protocol for adolescents. J Child Psychol Psychiatry 2008;49:915–23.
18. Nixon GM, Thompson JM, Han DY, et al. Short sleep duration in middle childhood: risk factors and consequences. Sleep 2008;31:71–8.
19. Talbot LS, McGlinchey EL, Kaplan KA, Dahl RE, Harvey AG. Sleep deprivation in adolescents and adults: changes in affect. Emotion 2010;10:831–41.
20. Baum KT, Desai A, Field J, Miller LE, Rausch J, Beebe DW. Sleep restriction worsens mood and emotion regulation in adolescents. J Child Psychol Psychiatry 2014;55:180–90.
21. Voderholzer U, Piosczyk H, Holz J, et al. Sleep restriction over several days does not affect long-term recall of declarative and procedural memories in adolescents. Sleep Med 2011;12:170–8.
22. Gradisar M, Terrill G, Johnston A, Douglas P. Adolescent sleep and working memory performance. Sleep Biol Rhythms 2008;6:146–54.
23. Sadeh A, Gruber R, Raviv A. Sleep, neurobehavioral functioning, and behavior problems in school-age children. Child Dev 2002;73:405–17.

24. Steenari MR, Vuontela V, Paavonen EJ, Carlson S, Fjallberg M, Aronen E. Working memory and sleep in 6- to 13-year-old schoolchildren. J Am Acad Child Adolesc Psychiatry 2003;42:85–92.
25. Buckhalt JA, El-Sheikh M, Keller P. Children's sleep and cognitive functioning: race and socioeconomic status as moderators of effects. Child Dev 2007;78:213–31.
26. Sadeh A, Gruber R, Raviv A. The effects of sleep restriction and extension on school-age children: what a difference an hour makes. Child Dev 2003;74:444–55.
27. Kopasz M, Loessl B, Valerius G, et al. No persisting effect of partial sleep curtailment on cognitive performance and declarative memory recall in adolescents. J Sleep Res 2010;19:71–9.
28. Beebe DW, Difrancesco MW, Tlustos SJ, McNally KA, Holland SK. Preliminary fMRI findings in experimentally sleep-restricted adolescents engaged in a working memory task. Behav Brain Funct 2009;5:9.
29. Astill RG, Van der Heijden KB, Van Ijzendoorn MH, Van Someren EJ. Sleep, cognition, and behavioral problems in school-age children: a century of research meta-analyzed. Psychol Bull 2012;138:1109–38.
30. Basner M, Dinges DF. Maximizing sensitivity of the Psychomotor Vigilance Test (PVT) to sleep loss. Sleep 2011;34:581–91.
31. Lo JC, Groeger JA, Santhi N, et al. Effects of partial and acute total sleep deprivation on performance across cognitive domains, individuals and circadian phase. PLoS One 2012;7:e45987.
32. Dinges DF, Powell JW. Microcomputer analyses of performance on a portable, simple visual RT task during sustained operations. Beh Res Meth Instr Comp 1985;17:652–5.
33. Lim J, Dinges DF. Sleep deprivation and vigilant attention. Ann N Y Acad Sci 2008;1129:305–22.
34. Buysse DJ, Reynolds CF III, Monk TH, Berman SR, Kupfer DJ. The Pittsburgh Sleep Quality Index: a new instrument for psychiatric practice and research. Psychiatry Res 1989;28:193–213.
35. Horne JA, Ostberg O. A self-assessment questionnaire to determine morningness-eveningness in human circadian rhythms. Int J Chronobiol 1976;4:97–110.
36. Meijer AM. Chronic sleep reduction, functioning at school and school achievement in preadolescents. J Sleep Res 2008;17:395–405.
37. Johns MW. A new method for measuring daytime sleepiness: the Epworth sleepiness scale. Sleep 1991;14:540–5.
38. Netzer NC, Stoohs RA, Netzer CM, Clark K, Strohl KP. Using the Berlin Questionnaire to identify patients at risk for the sleep apnea syndrome. Ann Intern Med 1999;131:485–91.
39. Beck AT, Steer RA. Beck Anxiety Inventory Manual. San Antonio, TX: Harcourt Brace and Company, 1993.
40. Beck AT, Steer RA, Brown GK. Manual for the Beck Depression Inventory-II. San Antonio. TX: Psychological Corporation, 1996.
41. Raven J. Advanced progressive matrices: set II (1962 revision). London: H. K. Lewis, 1978.
42. Akerstedt T, Gillberg M. Subjective and objective sleepiness in the active individual. Int J Neurosci 1990;52:29–37.
43. Robertson IH, Manly T, Andrade J, Baddeley BT, Yiend J. 'Oops!': performance correlates of everyday attentional failures in traumatic brain injured and normal subjects. Neuropsychologia 1997;35:747–58.
44. Smith A. Symbol Digit Modalities Test. Los Angeles, CA: Western Psychological Services, 1991.
45. Klein KE, Wegmann HM, Athanassenas G, Hohlweck H, Kuklinski P. Air operations and circadian performance rhythms. Aviat Space Env Med 1976;47:221–30.
46. Watson D, Clark LA, Tellegen A. Development and validation of brief measures of positive and negative affect: the PANAS scales. J Pers Soc Psychol 1988;54:1063–70.
47. Macmillan NA, Creelman CD. Detection theory: a user's guide. New York, NY: Cambridge University Press, 1991.
48. Pollack L, Norman DA. A non-parametric analysis of recognition experiments. Psychon Sci 1964;1:125–6.
49. Iber C, Ancoli-Israel S, Chesson A, Quan SF. The AASM manual for the scoring of sleep and associated events: rules, terminology, and technical specification, 1st ed. Westchester, IL: American Academy of Sleep Medicine, 2007.
50. Selya AS, Rose JS, Dierker LC, Hedeker D, Mermelstein RJ. A practical guide to calculating Cohen's f(2), a measure of local effect size, from PROC MIXED. Front Psychol 2012;3:111.
51. Cohen J. Statistical power analysis for the behavioral sciences, 2nd ed. Hillsdale, NJ: Lawrence Erlbaum Associates, 1988.
52. Belenky G, Wesensten NJ, Thorne DR, et al. Patterns of performance degradation and restoration during sleep restriction and subsequent recovery: a sleep dose-response study. J Sleep Res 2003;12:1–12.
53. Van Dongen HP, Maislin G, Mullington JM, Dinges DF. The cumulative cost of additional wakefulness: dose-response effects on neurobehavioral functions and sleep physiology from chronic sleep restriction and total sleep deprivation. Sleep 2003;26:117–26.
54. Steptoe A, Peacey V, Wardle J. Sleep duration and health in young adults. Arch Intern Med 2006;166:1689–92.
55. Matricciani L, Olds T, Petkov J. In search of lost sleep: secular trends in the sleep time of school-aged children and adolescents. Sleep Med Rev 2012;16:203–11.
56. Larson RW, Verma S. How children and adolescents spend time across the world: work, play, and developmental opportunities. Psychol Bull 1999;125:701–36.
57. Dahl RE. The impact of inadequate sleep on children's daytime cognitive function. Semin Pediatr Neurol 1996;3:44–50.
58. Chee MW, Chuah YM. Functional neuroimaging and behavioral correlates of capacity decline in visual short-term memory after sleep deprivation. Proc Natl Acad Sci U S A 2007;104:9487–92.
59. Chuah LY, Chee MW. Cholinergic augmentation modulates visual task performance in sleep-deprived young adults. J Neurosci 2008;28:11369–77.
60. Wee N, Asplund CL, Chee MW. Sleep deprivation accelerates delay-related loss of visual short-term memories without affecting precision. Sleep 2013;36:849–56.
61. Chee MW. Limitations on visual information processing in the sleep-deprived brain and their underlying mechanisms. Curr Opin Behav Sci 2015;1:56–63.
62. Tucker AM. Two independent sources of short term memory problems during sleep deprivation. Sleep 2013;36:815–7.
63. Banks S, Van Dongen HP, Maislin G, Dinges DF. Neurobehavioral dynamics following chronic sleep restriction: dose-response effects of one night for recovery. Sleep 2010;33:1013–26.
64. Rupp TL, Wesensten NJ, Balkin TJ. Trait-like vulnerability to total and partial sleep loss. Sleep 2012;35:1163–72.
65. Christakis NA, Fowler JH. Social contagion theory: examining dynamic social networks and human behavior. Stat Med 2013;32:556–77.
66. Stickgold R, Walker MP. Sleep-dependent memory triage: evolving generalization through selective processing. Nat Neurosci 2013;16:139–45.
67. Payne JD, Stickgold R, Swanberg K, Kensinger EA. Sleep preferentially enhances memory for emotional components of scenes. Psychol Sci 2008;19:781–8.
68. Sterpenich V, Albouy G, Boly M, et al. Sleep-related hippocampo-cortical interplay during emotional memory recollection. PLoS Biol 2007;5:e282.
69. Ancoli-Israel S, Cole R, Alessi C, Chambers M, Moorcroft W, Pollak CP. The role of actigraphy in the study of sleep and circadian rhythms. Sleep 2003;26:342–92.

ACKNOWLEDGMENTS

The authors are grateful for Hans Van Dongen for his advice on statistical analyses, and Sher Yi Chiam and Benny Chin Seah Koh for their assistance in participant recruitment in their schools. We thank Amiya Patanaik and Jasmine Siudzinski for coding the cognitive tasks, and Jesisca Tandi, Wei Shan Cher, Pearlynne Chong, Bindiya Lakshmi Raghunath, V Vien Lee,

Shin Wee Chong, and Nicholas Ivan Chee for their effort in data collection and processing.

SUBMISSION & CORRESPONDENCE INFORMATION

Submitted for publication August, 2015
Submitted in final revised form September, 2015
Accepted for publication October, 2015
Address correspondence to: Dr. Michael W.L. Chee, Centre for Cognitive Neuroscience, Duke-NUS Graduate Medical School, 8 College Road, Level 6, Singapore 169857; Tel: (+65) 6516 4916; Fax: (+65) 6221 8625; Email: michael.chee@duke-nus.edu.sg

DISCLOSURE STATEMENT

This was not an industry supported study. Financial support was provided by the National Medical Research Council, Singapore (NMRC/STaR/0004/2008 and NMRC/STaR/015/2013) and The Far East Organization. The authors have indicated no financial conflicts of interest. This work was approved by the Institutional Review Board of the National University of Singapore (13-562). All participants provided written informed consent.
